# Target delivery of a PD-1-TREM2 scFv by CAR-T cells enhances anti-tumor efficacy in colorectal cancer

**DOI:** 10.1186/s12943-023-01830-x

**Published:** 2023-08-10

**Authors:** Jian Chen, Tianchuan Zhu, Guanmin Jiang, Qi Zeng, Zhijian Li, Xi Huang

**Affiliations:** 1 Center for Infection and Immunity and Guangdong Provincial Engineering Research Center of Molecular Imaging, The Fifth Affiliated Hospital of Sun Yat-sen University, 519000 Zhuhai, Guangdong China; 2 Department of Clinical Laboratory, The Fifth Affiliated Hospital of Sun Yat-sen University, 519000 Zhuhai, Guangdong China; 3 Department of Oncology, The Fifth Affiliated Hospital of Sun Yat-sen University, 519000 Zhuhai, Guangdong China; 4 The Fourth People’s Hospital of Foshan, 528000 Foshan, Guangdong China

**Keywords:** CAR-T, TREM2, PD-1, Colorectal cancer, Tumor microenvironment, Single-chain variable fragment (scFv)

## Abstract

**Background:**

Chimeric antigen receptor (CAR) -T cell therapy is an efficient therapeutic strategy for specific hematologic malignancies. However, positive outcomes of this novel therapy in treating solid tumors are curtailed by the immunosuppressive tumor microenvironment (TME), wherein signaling of the checkpoint programmed death-1 (PD-1)/PD-L1 directly inhibits T-cell responses. Although checkpoint-targeted immunotherapy succeeds in increasing the number of T cells produced to control tumor growth, the desired effect is mitigated by the action of myeloid-derived suppressor cells (MDSCs) and tumor-associated macrophages (TAMs) in the TME. Previous studies have confirmed that targeting triggering-receptor-expressed on myeloid cells 2 (TREM2) on TAMs and MDSCs enhances the outcomes of anti-PD-1 immunotherapy.

**Methods:**

We constructed carcinoembryonic antigen (CEA)-specific CAR-T cells for colorectal cancer (CRC)-specific antigens with an autocrine PD-1-TREM2 single-chain variable fragment (scFv) to target the PD-1/PD-L1 pathway, MDSCs and TAMs.

**Results:**

We found that the PD-1-TREM2-targeting scFv inhibited the activation of the PD-1/PD-L1 pathway. In addition, these secreted scFvs blocked the binding of ligands to TREM2 receptors present on MDSCs and TAMs, reduced the proportion of MDSCs and TAMs, and enhanced T-cell effector function, thereby mitigating immune resistance in the TME. PD-1-TREM2 scFv-secreting CAR-T cells resulted in highly effective elimination of tumors compared to that achieved with PD-1 scFv-secreting CAR-T therapy in a subcutaneous CRC mouse model. Moreover, the PD-1-TREM2 scFv secreted by CAR-T cells remained localized within tumors and exhibited an extended half-life.

**Conclusions:**

Together, these results indicate that PD-1-TREM2 scFv-secreting CAR-T cells have strong potential as an effective therapy for CRC.

**Supplementary Information:**

The online version contains supplementary material available at 10.1186/s12943-023-01830-x.

## Background

Colorectal cancer (CRC) is the second most common cause of cancer-related deaths worldwide[1], with 1.9 million CRC cases and 576,000 CRC-related deaths reported in 2020 alone [2]. Globally, a consistent increase in the incidence of CRC and related deaths with increasing population age has been observed [3]. In addition to DNA mismatch repair and genetic hypermutability, genetic alterations leading to the accumulation of oncogenes, such as *APC, KRAS*, and *PIK3CA* are considered to be the main drivers of CRC tumorigenesis [4]. Surgical resection, chemotherapy, and radiation are the major clinical therapeutic modalities currently used for treating CRC. Unfortunately, patients with CRC have a poor prognosis, with an overall 5-year survival rate less than 15% [5]. Therefore, novel and advanced treatment options with improved therapeutic efficacy are urgently needed to increase the life expectancy and quality of life of patients with CRC.

In recent years, an increasing number of novel immunotherapies have been clinically applied based on exploiting the body’s immune system to combat tumors [6]. Chimeric antigen receptor (CAR)-T-cell therapy is considered one of the most effective immunotherapeutic approaches, particularly for treating hematologic malignancies [7]. As a result, anti-CD19 CAR-T cell therapy has been approved by the Food and Drug Administration (FDA) for clinical use in patients with refractory B-cell acute lymphoblastic leukemia [8]. However, the clinical potential of CAR-T cells in alleviating solid tumors has not been fully explored. The results of a Phase I clinical study (NCT02349724), employing carcino-embryonic antigen (CEA)-CAR-T therapy administered systemically through intravenous infusion to treat patients with CRC, revealed an absence of sustained therapeutic effects [9]. In addition to genetic and epigenetic manipulation, the occurrence and development of CRC are significantly influenced by the tumor microenvironment (TME), which comprises immune checkpoints such as programmed death1 (PD-1) / programmed death-ligand 1 (PD-L1), LAG-3, CTLA4, and TIM3 that infiltrate immunosuppressive myeloid cells and regulatory T cells, thereby suppressing endogenous anti-tumor immunity and the effects of CAR-T therapy [10]. Combination immunotherapy using CAR-T cells and immune checkpoint blockade (anti-PD-1) therapy results in an enhanced anti-tumor effect on both solid tumors and hematologic malignancies [11, 12]. However, targeting PD-1 pathway elicits a strong clinical response only in patients with microsatellite instability-high CRC, and less than 5% of patients respond to checkpoint blockade therapy [13], indicating additional involvement of the TME in mediating resistance to cancer immunotherapy. Therefore, several recent studies have focused on identifying novel molecules that target the TME and combination approaches that could expand the benefits of CAR-T immunotherapy [14].

Triggering receptor expressed on myeloid cells 2 (TREM2), a member of the immunoglobulin superfamily, binds lipids, transmits intracellular signals via the adaptor DNAX-activating protein of 12 kDa (DAP12) and plays a critical role in the pathogenesis of Alzheimer’s disease (AD) [15]. TREM2-activated microglia wrap and selectively disrupt the toxic protein aggregates that characterize AD [16]. In the TME, TREM2 plays a significant role in myeloid immunosuppression [17]. TREM2 is mainly expressed on the surface of tumor-associated macrophages (TAMs) and functions as an essential phenotypic marker for both TAMs and monocytes with potent immunosuppressive activity [18]. Targeting TREM2 on TAMs was shown to enhance the efficacy of immunotherapy for ovarian cancer [19]. Therefore, TREM2 is a potent therapeutic target directed against the TME. The absence of TREM2 enhances the anti-PD-1-mediated immunotherapy in CRC animal models, with a study revealing that MC38 tumor growth was significantly inhibited in *Trem2*^*–/–*^ mice compared to that in wild-type mice [20]. Therefore, a combination of antibodies against TREM2 and PD-1 holds potential for significant enhancement of CAR-T cell function in treating tumors.

The use of full-length antibodies in the development of tumor therapeutics is limited by their high molecular weight, low permeability in tissues, and toxicity associated with crystallizable fragment (Fc) segments[21]. In the present study, we aimed to develop a single therapy for CRC using CAR-T cell delivery of a combined PD-1-TREM2 single-chain fragment variable (scFv) derived from anti-TREM2 and anti-PD-1 antibodies designed for specific localization at the tumor site. Additionally, we aimed to prolong the half-life of the PD-1-TREM2 scFv to enhance its clinical applicability. We found that PD-1-TREM2 scFv exhibits improved tumor tissue penetration owing to its low molecular weight, although this scFv has a shorter half-life in the body than the full-length antibody. The PD-1-TREM2 scFv continuously secreted by CAR-T cells could target the TME, thereby remodeling its immunosuppression and enhancing the efficacy of CAR-T therapy in CRC.

## Materials and methods

### Collection of tumor tissues from patients

Colorectal cancer and corresponding normal tissue samples were collected at the Fifth Affiliated Hospital of Sun Yat-Sen University (China). The tissue samples were obtained with informed, written consent from the patients and the approval of the local ethics committee.

### Animals and cell lines

C57BL/6 mice were purchased from the Guangdong Medical Laboratory Animal Center and *Trem2–/–* animals were generated at the Model Animal Research Center (MARC) of Nanjing University (Nanjing, China). All animals were bred at under Guangdong Provincial Engineering Research Center of Molecular Imaging for cancer research. All experimental protocols were approved by the Ethics Committee Board for Human Experiments at the Fifth Affiliated Hospital of Sun Yat-Sen University. The murine CRC cell line MC38, the human embryonic kidney cell line HEK-293T, and mouse mononuclear macrophages cell line RAW 264.7 were obtained from the American Type Culture Collection (Manassas, VA, USA). All cells culture dishes/plates were purchased from NEST Biotechnology Co.Ltd.(Wuxi,China). All cell lines were cultured in Dulbecco’s modified Eagle medium (DMEM)/RPMI-1640 (Thermo Fisher Scientific, USA) containing 10% fetal bovine serum (Thermo Fisher Scientific, USA), 100 IU/ml penicillin (Sigma, USA), and 100 µg/ml streptomycin (Sigma, USA) at 37 °C with 5% CO_2_.

### Production of the bi-specific scFv antibody

The PD-1–TREM2 bi-specific scFv antibody (BsAb), PD-1-TREM2 scFv, capable of simultaneously targeting the PD-1 pathway and TREM2 on TAMs was constructed by linking the PD-1 scFv derived from the anti-mouse PD-1 monoclonal antibody RMP1-14 [22] and TREM2 scFv (patent application WO2020123664A [19]) with a flexible (glycine4-serine)3 linker. A polyhistidine-tag (His-tag) element was added to this construct for protein purification and detection, which was cloned into the pcDNA3.1 eukaryotic expression vector to produce the BsAb. Chinese hamster ovary (CHO) cells were used to express BsAb. Expression and purification of the BsAb were using i–nitrilotriacetic acid (NTA) agarose beads according to previously described protocols [22]. Sodium dodecyl sulfate-polyacrylamide gel electrophoresis (SDS-PAGE) analysis was performed to evaluate protein purity.

### Construction of CARs

The sequences of PD-1-scFv and TREM2-scFv were derived from the RPM1-14 antibody and patent application WO2020123664A1, respectively, with a His-tag element [23]. The sequences of CEA-4-1BB-containing CAR were designed according to previous reports [24, 25]. CART-CEA, and the PD-1-scFv, TREM2-scFv and BsAb linked with CEA-4-1-BB-CAR by the signal peptides (CART-CEA.sPD-1 scFv, CART-CEA.sTREM2 scFv, and CART-CEA.sBsAb, respectively) were then inserted into the pCDH-EF1 lentiviral vector containing a transgene encoding green fluorescent protein (GFP) as the fluorescent reporter. All sequences were synthesized by Genscript Company.

**Construction of MC38 cell with stable CEA expression and HEK-293T cells with stable expression of PD-1**.

The pCDH-EF1-CEA(NM_001037168.1)-luciferase sequence was synthesized by Genscript Company to produce MC38 cells with stable expression of CEA. The pCDH-EF1-PD-1(NM_001042451.2)-myc sequence was synthesized by Genscript Company to produce 293T cells with stable expression of PD-1.

HEK-293T cells transfected with three different plasmids: the target plasmid (pCDH-EF1-CEA-luciferase / pCDH-EF1-PD-1), psPAX2, and pMD2.G (Addgene, USA) and the supernatant was collected after 48 h. The lentiviral supernatant was concentrated using PEG-2000 (Sigma) following a previously described protocol [26]. MC38 cells/ HEK-293T cells were transfected with the concentrated lentiviral supernatant and screening with puromycin, respectively.

### Mouse T cell transduction and culture

T cells were derived from murine splenocytes using a magnetic bead-based selection method according to the manufacturers protocol (Solarbio, China and Stem cell, Canada). The cells were cultured in RPMI-1640 medium containing 10% fetal bovine serum, 100 IU/ml penicillin, and 100 µg/ml streptomycin at 37 °C with 5% CO_2_. T cells were stimulated overnight with mouse CD3/CD28 (Stem cell, Canada) antibodies and supplemented with recombinant mouse interleukin (IL-2) (Stem cell, Canada). Subsequently, the lentivirus and polybrene were added to the culture medium and mixture immediately. Fresh medium containing recombinant mouse IL-2 was added after 8 h. After infection, the medium was replaced every 2 or 3 days.

### Tumor models

Six-week-old healthy male C57BL/6 mice were used for all animal experiments. For establishment of the MC38 CRC tumor model, 2 × 10^6^ CEA-expressing MC38 cells were washed and resuspended in 100µL saline and then subcutaneously inoculated in mice. The mice were monitored every day and tumors were measured every other day for 23 days.

### In vivo treatment

To investigate the therapeutic effect of the BsAb, mice were treated intraperitoneally with PD-1-scFv, TREM2-scFv, and the BsAb at 200 µg/mouse every other day starting at 7 days after tumor cell injection. Mice were sacrificed on day 23 when tumors reached 1.5 cm in diameter.

To investigate the therapeutic effect of the CART-CEA-sBsAb, mice were intravenously infused with 1 × 10^7^ CAR-T cells (CART-CEA, CART-CEA.sPD-1 scFv, CART-CEA.sTREM2 scFv and CART-CEA.sBsAb) or with phosphate-buffered saline (PBS) as the control on day 7 after subcutaneous injection with 2 × 10^6^ CEA-MC38 cells. To monitor tumor growth, mice were anesthetized and intraperitoneally injected with 3 mg D-luciferin (Beyotime, China). After 15 min, the mice were imaged using an IVIS Imager. After CAR-T cells treatment, 100µL of venous blood was extracted every 5 days from the orbital venous plexus of the mice into 0.5 mL K_2_EDTA anticoagulation centrifuge tubes.

### Enzyme-linked immunosorbent assay (ELISA) for cytokine analysis

Mouse tumor supernatant samples were detected for intra-tumor cytokine levels using an ELISA kit (DAKEWE, China). Mouse tumor tissues were enzymatically dissociated, and then the tumor supernatant was collected and evaluated for intra-tumor cytokine levels (interferon (IFN)-γ, IL-2, tumor necrosis factor (TNF)-α and IL-15) using respective ELISA kits (DAKEWE, China) according to the manufacturer’s instructions.

The binding ability of PD-1 scFv, TREM2 scFv, and bi-specific scFv to PD-1 and TREM2 protein were evaluated by sandwich ELISA, wherein recombinant mouse PD-1-Fc (GenScript) or TREM2 protein (R&D Systems) (200ng/mL) were coated on the plates, followed by purified PD-1 scFv, TREM2 scFv, and bi-specific scFv detected by His-tag antibody-HRP (1:500 diluted; Abcam).

IFN-γ, Granzyme B, and Perforin secreted by engineered CAR-T cells were measured by the mouse IFN-γ, Granzyme B, and Perforin ELISA kits (DAKEWE, China). Briefly, 1 × 10^6^ engineered CAR-T cells were co-cultured with 1 × 10^6^ MC38-CEA cells per well in a 96-well round-bottom plate in a 200µL medium for 48 h. The concentration of IFN-γ, Granzyme B, and Perforin in the supernatants were determined by following the manufacturer’s protocol.

### Flow cytometry

To obtain single-cell suspensions, the mouse tumor tissues were minced and digested, and then filtered through 70μm strainers. Erythrocytes in the cell suspension were lysed by RBC lysis buffer (Biolegend, USA), and the supernatant was removed by centrifugation. Cells were washed with PBS and stained for flow cytometry with the following antibodies: CD3-APC, CD45-PE-cy7/FITC, CD4-FITC, CD8-PE/pecy7, CD11b-PE, GR1-PC5.5, F4/80-APC, CD206-FITC, His-APC, Ly6G-PC5.5, TREM2-PE, PD-1-FITC, IFN-γ-PE, PE Goat anti-mouse IgG, Granzyme B-PE, and Perforin-PE (BioLegend).

### Immunohistochemistry and immunofluorescence staining

Paraffin-embedded normal and tumor tissues were dewaxed, dehydrated, antigenically fixed, and sliced into 4 μm-thick sections for immunohistochemical staining. The sections were incubated with anti-human TREM2 antibody (Clone #237,920, 1:500, R&D) and anti-human CD206 antibody (#24,595, 1:1000, Cell Signaling Technology) overnight at 4 °C, followed by incubation with goat anti-rabbit secondary antibody (BOSTER), and finally stained with DAB (BOSTER) for visualization. DAPI (Abmole, USA) was used for staining of cell nuclei and blocking of sections prior to observation.

### Immunoprecipitation

HEK-293T cells with stable expression of PD-1 were co-incubated with PD-1 scFv, TREM2 scFv and BsAb at 200 µg/mL for 24 h. RAW 264.7 cells were co-incubated with PD-1 scFv, TREM2 scFv, and BsAb at 200 µg/mL for 24 h. Cellular lysates were prepared by incubating the cells in cell lysis buffer in the presence of the protease inhibitor phenylmethylsulfonyl fluoride (PMSF) for 30 min on ice, followed by centrifugation at 13,000 rpm for 10 min at 4 °C. For immunoprecipitation, cellular lysates were incubated with anti-His (Sigma-Aldrich), or consecutively incubated with PD-1(AB2, Sigma) or TREM-2 (clone E4J7A, CST) antibody and protein A/G agarose beads (EMD Millipore) for 12 h at 4 °C with constant rotation. The beads were then washed eight times using the cell lysis buffer. Between washes, the beads were collected by centrifugation at 13,000 rpm for 1 min at 4 °C. The precipitated proteins were eluted from the beads by resuspending the beads in 5× SDS- PAGE loading buffer and boiling for 10 min. The boiled immune complexes were subjected to SDS-PAGE, followed by immunoblotting with appropriate antibodies.

### Mouse primary cell culture

Mice were euthanized by breaking the neck, soaked in 75% alcohol for 2–3 s, and the superior hepatic segment of the inferior vena cava was ligated and entered at the level of the inferior vena cava needle. Open perfusion and make a small incision in the portal vein. Mouse primary hepatocytes were isolated by perfusion of livers using liberase (ThermoFisher, USA) in EBSS (Sigma-Aldrich, USA). The colon was rinsed repeatedly with the HBSS buffer, the intestinal contents were removed, and the colon was turned over and rinsed three times. The colon was placed in the conical flask and placed at 4 degrees for 1 h, and then changed into the new HBSS buffer, shaking violently and finally filtered and centrifuged to obtain the cell suspension. The obtained cells were cultured in DMEM containing 10% fetal bovine serum, 100 IU/mL penicillin, and 100 µg/mL streptomycin at 37 °C with 5% CO_2_.

### Cytotoxicity assay and cell lysis assays

A lactate dehydrogenase (LDH) release assay was performed to evaluate the cytotoxic effect of CAR-T cells on tumor cells. The MC38 cell suspension was incubated in a 96-well plate (100µL/well), and the plate was pre-incubated for 24 h. The plate was then incubated with different proportions of CAR-T cells for 6 h. Subsequently, 50µL of the supernatant was aspirated into a new 96-well plate and further incubated for 30 min at room temperature in the dark. Next, 50µL of stop solution was added to each well. The absorbance at 490 nm was measured immediately using an enzyme calibrator. The effects of anti-PD-1 scFv, anti-TREM2 scFv, and BsAb on the viability of mouse hepatocytes and intestinal epithelial cells were assessed by CCK-8 Cell Counting Kit (MCE, USA), according to the manufacturer’s protocol.

Cell lysis was evaluated using a chromium-51 (Cr-51) release assay. MC38 cells were loaded with 100µCi Cr51 and co-cultured with the engineered CAR-T cells were plated in a serial dilution to generate a spectrum of effector-to-target ratios. After 24 h of incubation at 37 °C, the release of free Cr^51^ was measured by the TopCount NXT system (PerkinElmer Inc. MA, USA) as previously described [27]. The proportion of lysis was calculated using the following formula: specific lysis (%) = 100 × (experimental Cr^51^cpm release – spontaneous Cr^51^cpm release)/ (total Cr^51^cpm release – spontaneous Cr51cpm release). All data are presented as the mean (± standard deviation) of triplicate wells.

### Quantitative real-time polymerase chain reaction (RT-PCR)

Total RNA was extracted from normal or tumor tissues and peripheral blood mononuclear cells using TRIzol reagent (Invitrogen, USA), according to the manufacturer’s protocol. The total RNA concentration was measured with a NanoDrop spectrophotomer. The RNA was reverse-transcribed to cDNA by the RevertAid First Strand cDNA Synthesis Kit (ThermoFisher Scientific). The RT-PCR mixture contained cDNA, SYBR Green, PCR Master Mix (Applied Biosystems, USA), and the primer sequences. The primers for analysis of tissue samples were as follows: human TREM2: 5’-GCACAGCCATCACAGACG-3’ (forward) and 5’-CACCTCCACCAGGACCTTC − 3’ (reverse); human β-actin: 5’- GCTCCTCCTGAGCGCAAG − 3’ (forward) and 5’- CATCTGCTGGAAGGTGGACA − 3’ (reverse). The results were calculated as the fold change in expression relative to the reference obtained using the 2^-ΔΔCt method.

CAR expression in the blood samples was detected using primers targeting the mouse CD8 a chain and 4-1BB, the portion of CAR and calculate the copy numbers. Copy numbers were calculated as previously described [28].

The following are the sequences of the primers used to detect CAR:

CAR forward: 5’- GTCCTTCTCCTGTCACTGGT − 3’; CAR reverse: 5’- CCTCTTCTTCCTGAGGGCAT − 3’.

### Western blotting assay and SDS-PAGE

Cells were added the cell lysis mixture containing the protease inhibitor PMSF on ice for 30 min and the total protein concentration was determined with a BCA kit (Thermo Fisher Scientific, USA). Western blotting assays were conducted as previously described [29] using anti-His primary secondary Antibodies and goat anti-rabbit (Abcam, UK) secondary antibody. The bands were visualized on a GE ImageQuant LAS 500 system. The purified anti-PD-1 scFv, anti-TREM2 scFv and BsAb were subjected to SDS-PAGE, followed by staining with Kaomas Brilliant Blue (MCE, USA).

### Hematoxylin and eosin (HE) staining

The main organs of mice, including the heart, liver, spleen, lung and kidney, were collected, fixed in formalin, soaked in 10% neutral buffered formalin, paraffin- embedded, and stained for HE according to a standard protocol [29]. Sections were examined for pathological changes under a light microscope.

### Statistical analysis

Statistical analyses were performed using unpaired Student’s t-test, analysis of variance, and the Kaplan-Meier method as appropriate; *p* ≤ 0.05 was considered statistically significant.

## Results

### TREM2 is associated with TAMs and prognosis in patients with CRC

**Fig. 1:**
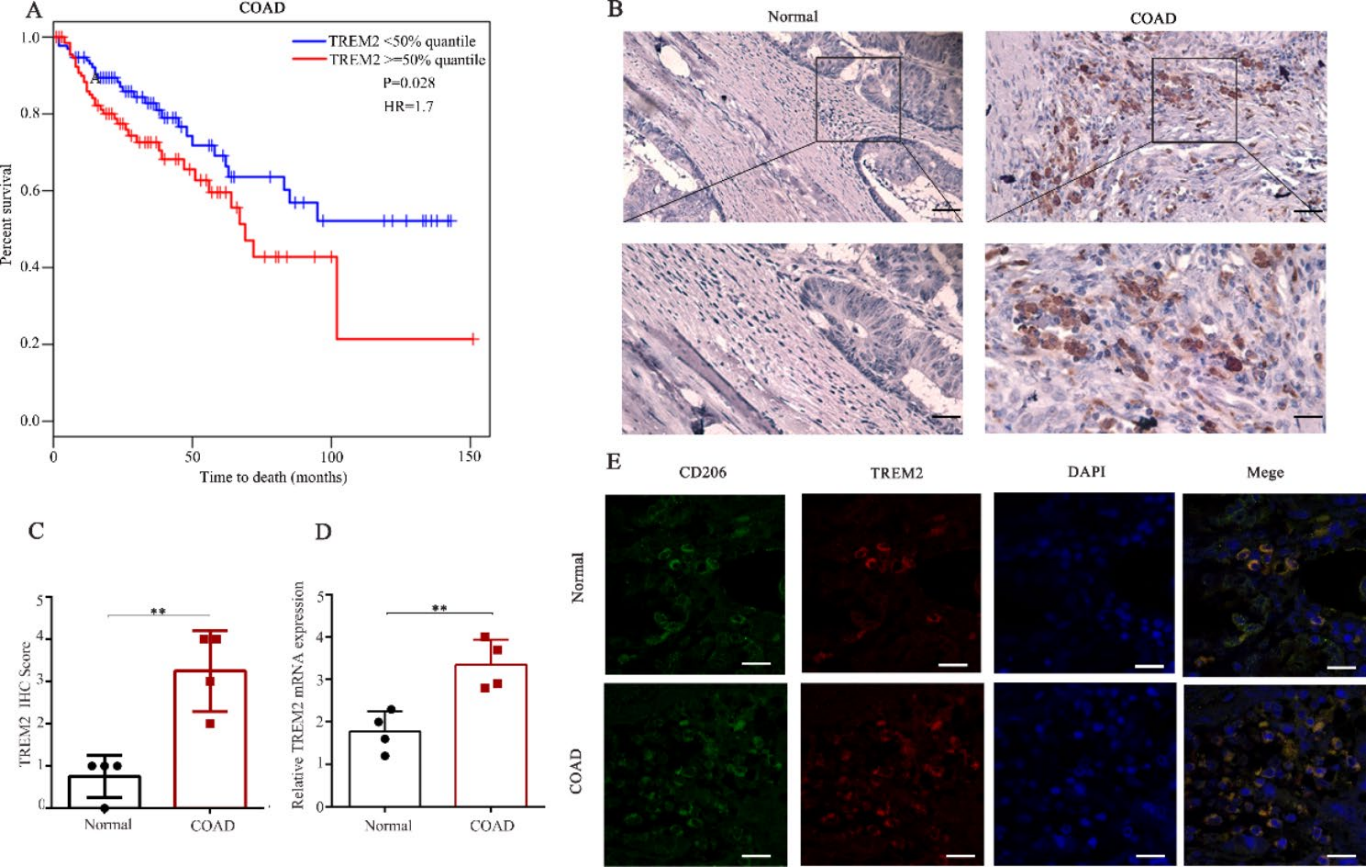
TREM2 is a potential marker of colorectal cancer (CRC). **(A)** Kaplan-Meier analysis representing the association of TREM2 expression with overall survival in CRC patients from The Cancer Genome Atlas cohort; ≥ 50% quantile of TREM2 expression is defined as the high-expressing group, and others are considered in the low-expressing group. **(B)** Immunohistochemistry staining representing TREM2 (brown) expression in CRC tissues. **(C)** Bar plots summarizing the immunohistochemistry semi-quantitative analysis; score 1, ≤ 25%; 2, 26–50%; 3, 51–75%; 4, > 75%. Scale bar: 100 μm; magnification: 200× (n = 4). **(D)** Relative TREM2 mRNA levels in CRC patients (n = 4). (**E**) CRC tissues or normal tissues were double stained with anti-CD206 (marked macrophages) (green) and anti-TREM2 (red) antibodies, and then observed by fluorescent microscopy. DAPI, blue (n = 10). Scale bars: 20 μm. Data are expressed as means ± SD; * *P* < 0.05, **P < 0.01, by unpaired Student’s t test (**C**-**D**). TREM2, triggering-receptor-expressed on myeloid cells 2; CD, cluster of differentiation; DAPI, 4′,6-diamidino-2-phenylindole

The Cancer Genome Atlas (TCGA) database was used to explore the correlation between TREM2 expression and clinical outcome in patients with CRC using a threshold of 50% quantile as the cut-off for dividing patients into high- and low-expression groups. A significant correlation was detected between enhanced TREM2 expression and decreased overall survival in the CRC group, indicating a direct association between TREM2 and CRC progression (Fig. 1A). Immunohistochemistry analysis revealed enhanced TREM2 expression in a significant number of cells in the tumor tissues of patients with CRC compared to that in resected normal specimens (Fig. 1B-C). Consistent with these above results, TREM2 mRNA expression levels were significantly enhanced in CRC patients compared with those of normal tissue controls, suggesting a prominent role of TREM2 in CRC pathogenesis (Fig. 1D). Further, confocal imaging revealed TREM2 and CD206-positive M2 TAMs co-localized in the CRC group more than in the control group (Fig. 1E). Taken together, these results suggested TREM2 as a potential therapeutic target of the CRC TME.

**Scheme 1:**
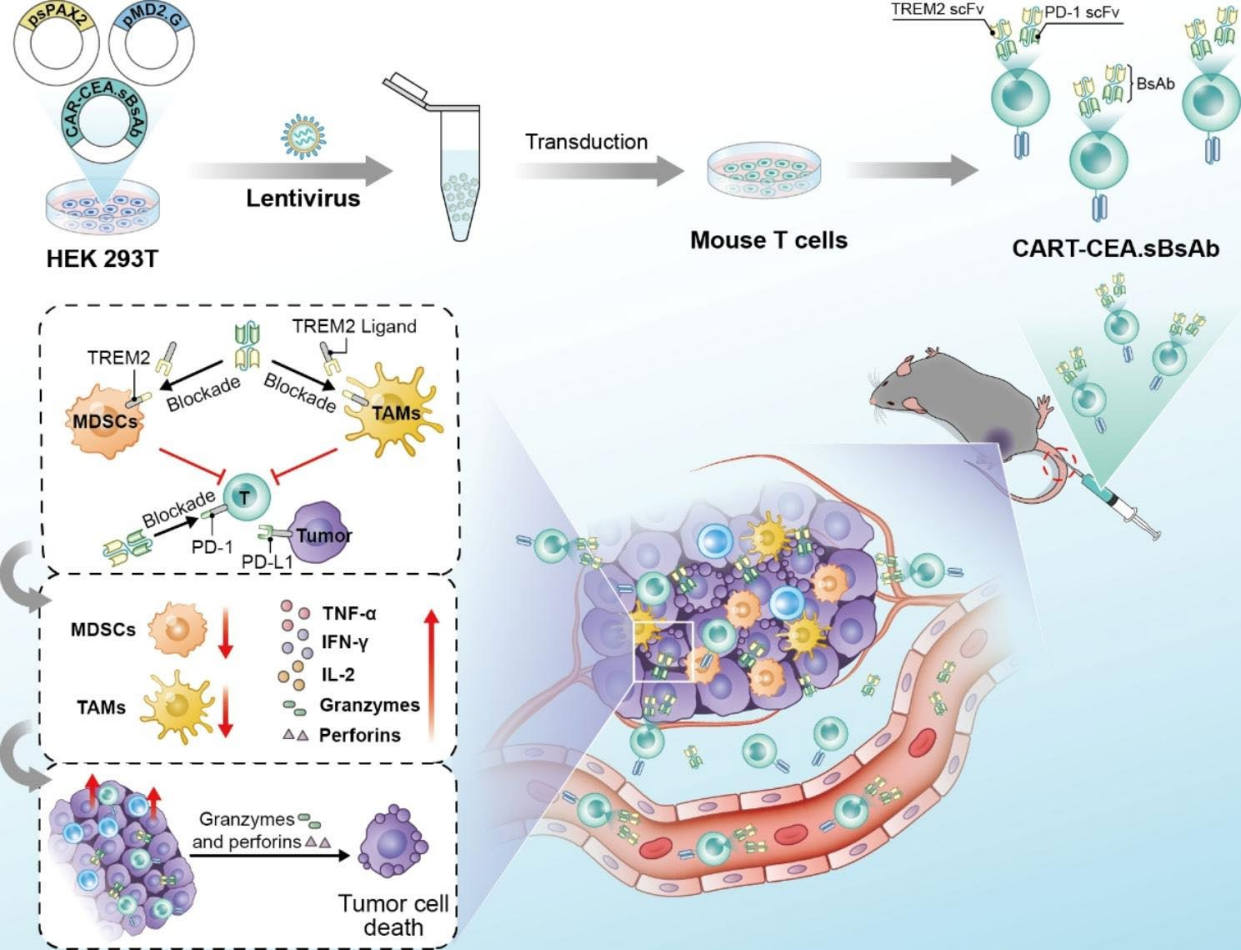
Schematic illustration of targeted delivery of a PD1-TREM2 scFv by CAR-T cells to enhances anti-tumor efficacy in colorectal cancer. First, the lentivirus encoding CART-CEA.sBsAb was generated using a three-plasmid lentiviral packing system. Mouse T cells were transduced with the encoding CART-CEA.sBsAb, and the successful preparation of autocrine PD-1-TREM2 scFv CAR-T cells (CART-CEA.sBsAb) was achieved. The anti-tumor mechanism of CART-CEA.sBsAb was evaluated in a subcutaneous CRC mouse model. The PD-1-TREM2 scFv secreted by CAR-T cells remained localized within tumors and blocked the PD-1/PD-L1 pathway and the binding of ligands to TREM2 receptors present on MDSCs and TAMs; the percentages of MDSCs and TAMs were decreased, and the levels of TNF-α, IFN-γ, IL-2, granzyme B, and perforin were enhanced. Finally, the intratumoral proportion and cytotoxic ability of CART-CEA.sBsAb increased

### Generation and characterization of a bi-specific scFv targeting PD-1 and TREM2

**Fig. 2:**
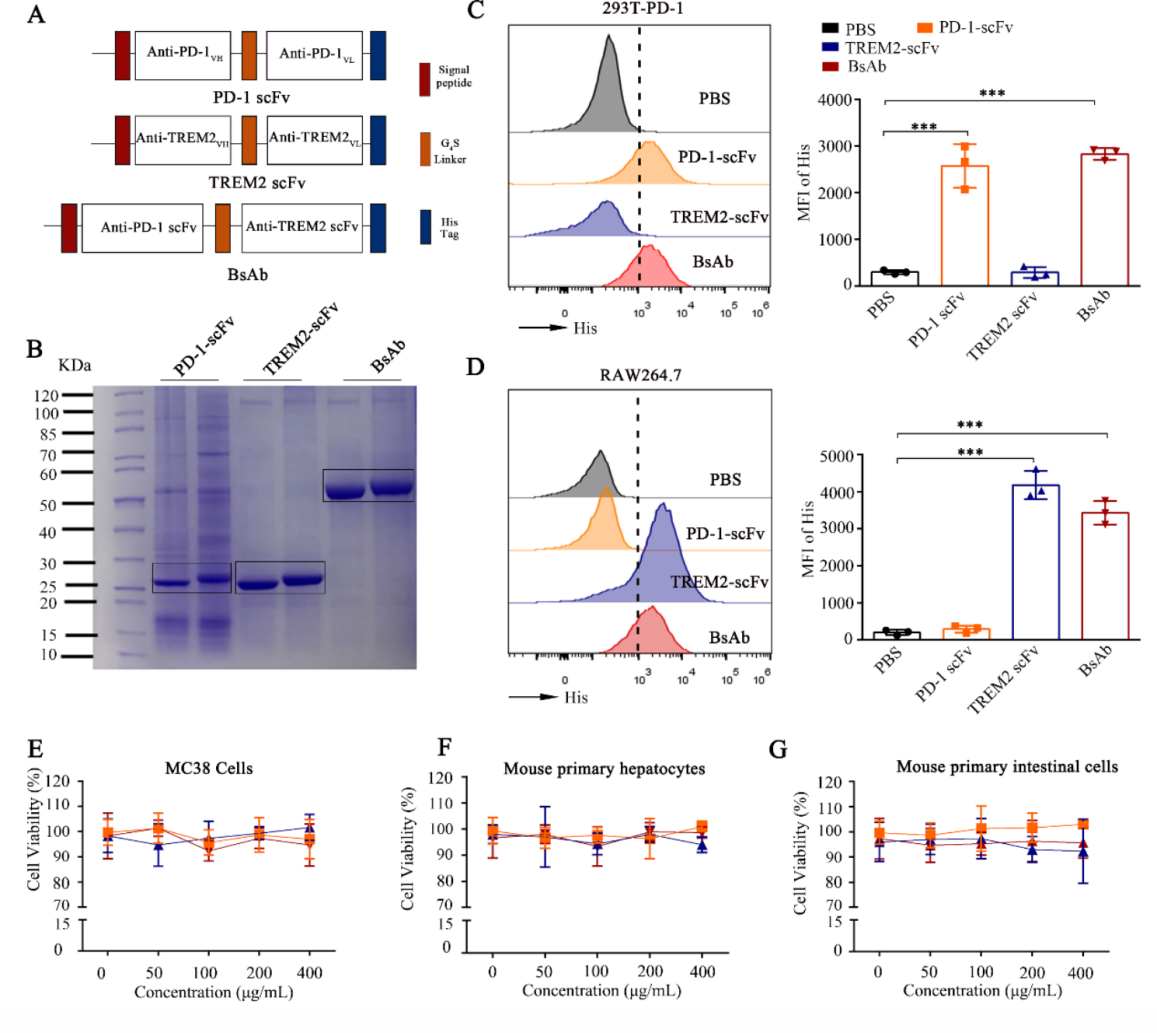
Construction and characterization of the bi-specific scFv antibody (BsAb). **(A)** Schematic representation of PD-1-scFv, TREM2-scFv, and BsAb. **(B)** SDS-PAGE analysis of PD-1 scFv, TREM2 scFv, and BsAb derived from the supernatants of CHO cells using nickel column purification and concentration. **(C)** Flow cytometric histograms demonstrating His-tag detection of PD-1 scFv, TREM2 scFv, and BsAb binding to 293T stably expressing PD-1 (293T-PD-1). The 293T-PD-1 cells were treated with PD-1 scFv, TREM2 scFv, and BsAb at 200 µg/mL and MFI of His was detected by flow cytometry. Bar plots summarizing the data are shown on the right. **(D)** Flow cytometric histograms demonstrating His-tag detection of PD-1 scFv, TREM2 scFv, and BsAb binding to TREM2 on the RAW 264.7 cells. PD-1 scFv, TREM2 scFv, and BsAb at 200 µg/mL and MFI of His was detected by flow cytometry. Bar plots summarizing the data are shown on the right. **(E-F)** Relative viability of MC38 cells, mouse primary hepatocytes, and mouse primary intestinal cells treated with PD-1 scFv, TREM2 scFv, and BsAb at 200 µg/mL for 24 h analyzed using the CCK-8 kit. Data are expressed as means ± SD; * *P* < 0.05, **P < 0.01 (n = 3), by unpaired Student’s t test (**C**-**D**). PD-1, programmed death-1; scFv, single-chain fragment variable; TREM2, triggering-receptor-expressed on myeloid cells 2; SDS-PAGE, sodium dodecyl-sulfate polyacrylamide gel electrophoresis; CHO, Chinese hamster ovary; RAW 264.7, Mouse Mononuclear Macrophages Cells; 293T, human embryonic kidneys; CCK, cell counting kit; SD, standard deviation

The 1608-bp BsAb sequence harboring two restriction sites on the pcDNA3.1 vector was expressed in CHO cells and purified using NTA (Fig. 2A). With reference to the ProtParam database, the molecular weight of the scFvs was predicted to be 26 and 56 kDa, respectively, as confirmed by SDS-PAGE (Fig. 2B).

The cell binding properties of PD-1-scFv, TREM2-scFv, and BsAb were analyzed using flow cytometry to detect the His-tag expression of 293T-PD-1 cells and the TREM2 expression of RAW 264.7 cells. 293T-PD-1 and RAW 264.7 cells were treated with PD1-scFv, TREM2-scFv, and BsAb and cultured for 24 h. The His detection was enhanced (Fig. 2C-D) in cultures treated with TREM2-scFv/BsAb and PD-1-scFv/BsAb, respectively, confirming the binding of BsAb on cell surfaces expressing PD-1 and TREM2. We also used CO-immunoprecipitation experiments and ELISA experiments further confirmed the binding of PD-1 scFv, TREM2 scFv and BsAb to PD-1/TREM2 molecules **(Fig. S1A-D)**.

The tumoricidal effect of the constructs was detected by analyzing cell viability using the CCK8 kit in mouse colon cancer MC38 cells cultured for 24 h with PD-1 scFv, TREM2 scFv, and BsAb. The results indicated the absence of a direct tumoricidal effect of PD-1 scFv, TREM2 scFv, and BsAb in MC38 cells (Fig. 2E). The scFvs were also found to be nontoxic in mouse primary hepatocytes and intestinal cells (Fig. 2F-G **and Fig S1E-F)**, thereby confirming the absence of nonspecific/side-effects in normal tissue cells in vitro.

### Anti-tumor efficacy of PD-1-TREM2 scFv in ***vivo*** using a CRC mouse model

**Fig. 3:**
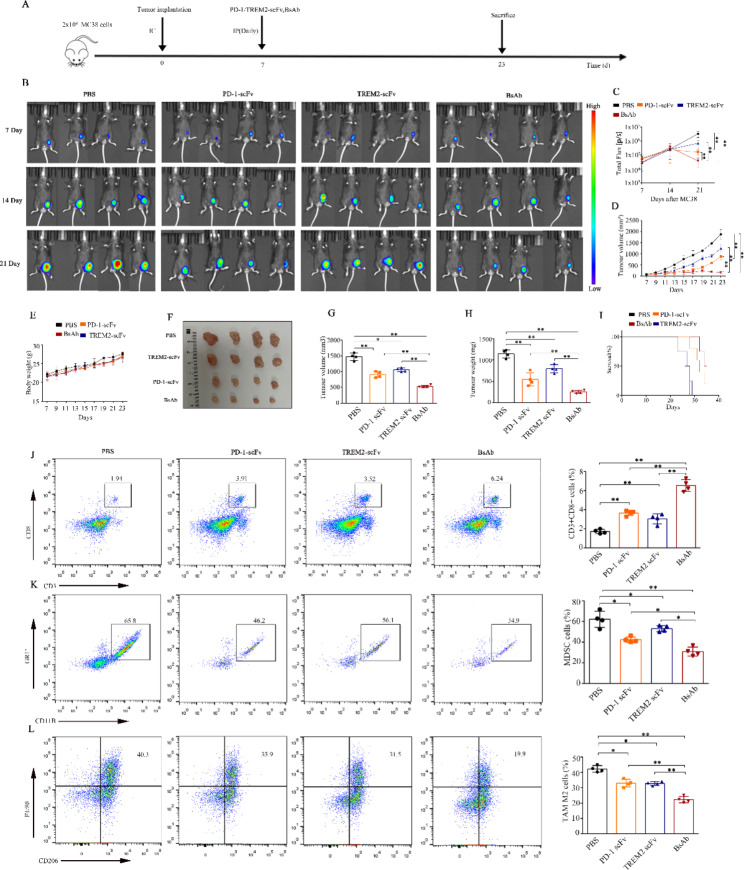
The bi-specific scFv antibody (BsAb) delays tumor growth and modulates intratumoral cytokines and the proportions of infiltrating immune cells, MDSCs, and M2 TAMs. **(A)** Schematic representation of the animal experiment. Six-week-old C57BL/6J mice were subcutaneously injected with 2 × 10^6^ MC38 cells expressing CEA. On day 7, mice received intravenous injection of PD-1 scFv, TREM2 scFv, or the BsAb at 200 µg/mouse daily; the PBS-treated group served as the control. **(B)** Bioluminescence images of mice treated with PD-1 scFv, TREM2 scFv, and BsAb at days 7, 14, and 21 (n = 4). **(C)** Bioluminescence signals of individual groups at day 7, 14, and 21 are shown on the Y-axis. The PBS group is indicated as black, the PD-1 scFv group is in orange, the TREM2 scFv group is in blue, and the BsAb group is in red. **(D-E)** Tumor volume and body weight of mice subjected to the indicated treatments (n = 4). **(F-H)** MC38-CEA tumors excised from individual groups of sacrificed mice on day 23. Tumor volume and weight of mice subjected to the indicated treatments (n = 4). **(I)** Effect of different treatments on the survival of tumor-bearing mice. **(J-L)** Effect of different treatment modalities on the infiltration of CD8-positive T cells (**J**), MDSCs (**K**), and M2 TAMs **(L)**. Proportions of tumor-infiltrating CD8-positive T cells, MDSCs, and M2 TAMs were detected by flow cytometry on day 23. All cells gated on CD45, CD45 + CD11b Gr1 + were marked as MDSCs, whereas cells gated at CD45 + CD11b F4/80 CD206 were marked as M2 TAMs. Representative plots are shown on the right (n = 4). Data are expressed as means ± SD; * *P* < 0.05; **P < 0.01, by unpaired Student’s t test and one-way ANOVA. scFv, single-chain fragment variable; CEA, carcinoembryonic antigen; PD-1, programmed death-1; TREM2, triggering-receptor-expressed on myeloid cells 2; PBS, phosphate buffered saline; MDSCs, myeloid-derived suppressor cells; TAMs, tumor associated macrophages; CD, cluster of differentiation

Having confirmed the activities of PD-1 scFv, TREM2 scFv, and BsAb in vitro, the anti-tumor efficacy of the BsAb was further analyzed in a CRC mouse models in vivo. The constructed MC38 cell line stably expressing mouse CEA **(Fig. S2A-B)** was used in the animal experiments as outlined in Fig. 3A. Significantly stronger and rapid inhibition of tumor progression was observed in BsAb-treated CRC mice compared with that in PD-1 scFv-, TREM2 scFv-, and PBS-treated CRC mice, as indicated by fluorescence intensity measurements (Fig. 3B-C). The tumor growth curve showed a trend consistent with the longitudinal bioluminescence assay, indicating significant early-stage inhibition of tumor growth in the BsAb-treated group compared with that in the PBS-, PD-1 scFv- and TREM2 scFv-treated groups (Fig. 3D **and Fig. S3A-D)**. Moreover, none of the tumor-bearing mice in all groups exhibited weight loss during the treatment period (Fig. 3E). Analysis of the anti-tumor efficacy of the BsAb in tumors excised from mice sacrificed on day 23 revealed a significant reduction in tumor volume and tumor weight in the BsAb-treated group compared with that in other groups (Fig. 3F-H). The survival of tumor-bearing mice was also significantly enhanced in the BsAb-treated mice compared with that in the other treatment groups (Fig. 3I), confirming the enhanced anti-tumor efficacy of BsAb in vivo.

Analysis of the BsAb treatment-induced in vivo immune response targeting the TME revealed enhanced infiltration of CD8-positive T cells (Fig. 3J) and a reduced percentage of myeloid-derived suppressor cells (MDSCs) and M2 TAMs (Fig. 3K-L) in the tumor specimens of BsAb-treated mice compared with those in tumors from the PD-1 scFv-, TREM2 scFv-, and PBS-treated groups. The release of perforin and granzyme B by cytotoxic CD8-positive T cells was significantly enhanced in the BsAb-treated group compared with that in the other treatment groups, as indicated by flow cytometry **(Fig. S4A-C)**. ELISA demonstrated enhanced levels of the inflammatory factors IL-2, IL-15, TNF-α, and IFN-γ in the intratumoral TME in the BsAb-treated group compared with those in the other groups **(Fig. S4D-G).** In line with these results, enhanced infiltration of CD8-positive T cells and reduced percentages of MDSCs and M2 TAMs were found in the TME of the analyzed tumor specimens in the PD-1 scFv- and TREM2 scFv-treated groups. Thus, the effect of PD-1-scFv and TREM2-scFv was minimal compared with that of BsAb PD-1-TREM2 scFv. Taken together, these results indicated that the BsAb enhanced the infiltration and cytotoxic ability of CD8-positive T cells in the tumor tissue by targeting MDSCs and M2 TAMs to overcome the inhibitory immune response of the TME.

To further verify whether the tumor will recur after PD-1 scFv, TREM2 scFv, and BsAb treatment, we extended the cycle of oncology treatment to 35 days. As shown in **Fig. S5A-C**), treatment with PD-1/TREM2 scFv and the BsAb did not completely prevent the possibility of tumor recurrence; the tumor recurred at approximately 21–28 days after treatment. Therefore, BsAb-treatment could not completely inhibit tumor growth and recurrence, suggesting the need to combine the BsAb with other treatment modalities for an effective therapeutic response.

### Construction and characterization of CAR-T cells secreting the BsAb

**Fig. 4:**
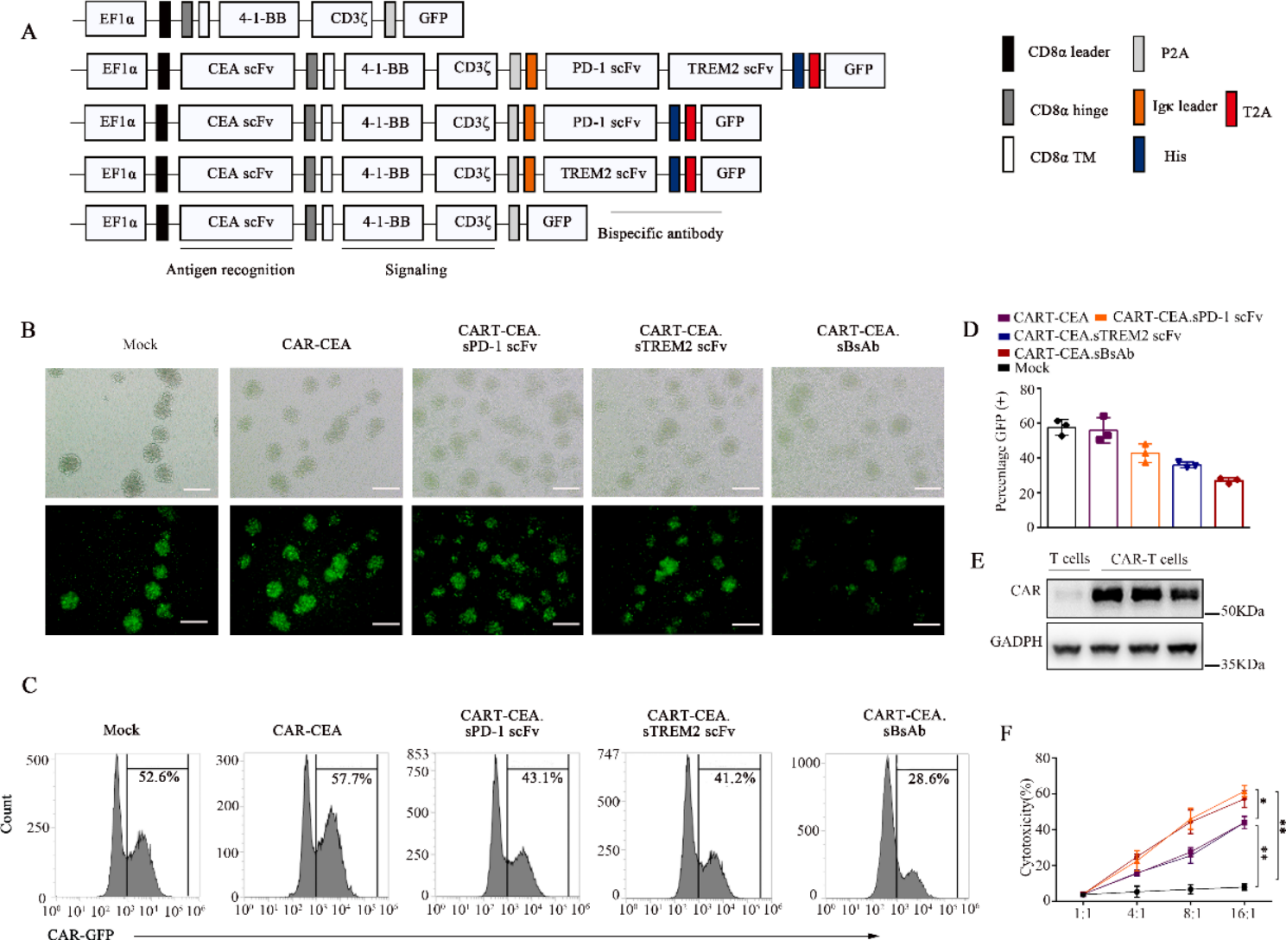
Construction and characterization of bi-specific scFv antibody (BsAb)- secreting CAR-T cells. (**A**) Schematic representation of Mock, PD-1 scFv, TREM2 scFv, and BsAb-secreting CAR constructs targeting CEA. CART-CEA was constructed as a control. (**B**) Efficient transduction of all constructs in mouse primary T cells as observed by fluorescence microscopy. Scale bar: 50 μm. (**C, D**) Flow cytometric assay analysis (**C**) and bar plot summary (**D**) indicating the transduction efficiency of all constructs in mouse primary T cells. (**E**) Western blot detecting the His-tag for CAR protein in mouse primary T cells. (**F**) Cytotoxicity of all constructed CAR-T cells against MC38 cells. All groups of CAR-T cells were co-cultured with MC38 cells at 1:1, 4:1, 8:1, and 16:1, ratios. MC38 cell lysis was detected by an LDH cytotoxicity kit. Data are expressed as means ± SD; * *P* < 0.05; **P < 0.01, by one-way ANOVA. CAR-T, chimeric antigen receptor-modified-T; PD-1, programmed death-1; scFv, single-chain fragment variable; TREM2, triggering-receptor-expressed on myeloid cells 2; CEA, carcinoembryonic antigen; LDH, lactate dehydrogenase

The experiments described above confirmed the strong anti-tumor efficacy of the BsAb in CRC mouse models. However, the BsAb has some disadvantages, such as a short half-life and the lack of tumor-targeted delivery[21]. Therefore, we combined the BsAb with CAR-T cells to overcome these limitations. Figure 4 A and **Table 1** provide a schematic representation and list of the viral vector constructs used in the study, respectively. The design was based on constructing a second-generation anti-CEA CAR containing anti-mouse CEA, a hinge and transmembrane domain, an intracellular 4-1BB costimulatory domain, and a CD3ζ activation domain. In the present study, the anti-CEA CAR was generated for secretion of anti-PD1 scFv, anti-TREM2 scFv, and PD-1-TREM2 scFv (BsAb) using a P2A element as the linker between the CEA CAR and secretion sequences, followed by a His-tag element for detection and purification. All vectors were incorporated with a (GFP) fluorescent reporter gene for we valuation of the transduction efficiency.

T cells isolated from mouse splenocytes were activated and transduced with each of the four CAR constructs using lentiviral vectors (Fig. 4B). Flowcytometry-based analysis of GFP expression was used to measure the success rate and efficacy of CAR transduction. Accordingly, the transduction efficiency of the Mock, PD-1 scFv, TREM2 scFv, and BsAb CAR constructs was approximately 53%, 58%, 43%, 41% and 30%, respectively (Fig. 4C-D). Consistent with the prediction, a 60-kDa protein was detected in the western blot of T cell samples isolated from transduced mice, confirming the successful construction of CARs (Fig. 4E). Secretion of PD-1 scFv, TREM2 scFv, and BsAb by mouse CAR-T cells was confirmed by detection of the respective proteins with SDS-PAGE of the concentrated supernatants **(Fig. S7A)**. The concentration of the secreted proteins in the supernatant increased with time, as detected by ELISA **(Fig. S7B)**. In addition, the protein activity was consistent with levels of secreted proteins **(Fig. S7C-D)**.

In line with a previous study showing that MDSCs can inhibit the proliferation and response of T cells [30], we also found that the BsAb targeting TREM2 on MDSCs could overcome the inhibitory immune response of the TME. To confirm whether CAR-CEA.sBsAb can reduce the inhibitory effect of MDSCs on CAR-T proliferation and survival, we co-cultured engineered CAR-T cells with MDSCs at a 1:1 ratio. As shown in **Fig. S6B and S6F**, the MDSCs inhibited the survival and proliferation of engineered CAR-T cells compared with those of engineered CAR-T cells cultured alone; the survival and proliferation of CAR-CEA.sPD-1, CAR-CEA.sTREM2, and CAR-CEA.sBsAb cells were greater than those of CAR-CEA cells. Moreover, survival and proliferation ability of CAR-T cells were higher under co-culture with TREM2-knockout MDSCs compared with those of CAR-T cells under co-culture with wild-type MDSCs. There results suggested that targeting TREM2 on MDSCs can alleviate the inhibitory effect of MDSCs to CAR-T cells **(Fig. S6F-G).**

Further, the tumoricidal efficacy of the engineered CAR-T cells was analyzed in vitro. Co-culture of MC38 cells at an effector to target ratio of 1, 4, 8, and 16 exhibited significantly enhanced tumoricidal activity in CART-CEA.sPD-1 and CART-CEA.sBsAb cells compared with that in CART-CEA and CART-CEA.sTREM2 cells (Fig. 4F **and Fig. S6A)**.The release of perforin, granzyme B, and IFN-γ by CAR-T cells was significantly enhanced in the CART-CEA.sPD-1 and CART-CEA.sBsAb groups compared with that in the other groups, as indicated by ELISA **(Fig. S6C-E)**. Thus, these results confirmed the successful construction of the engineered CAR-T cells.

### BsAb-secreting CAR-T cells exhibited enhanced anti-tumor function in the CRC model in vivo

**Fig. 5:**
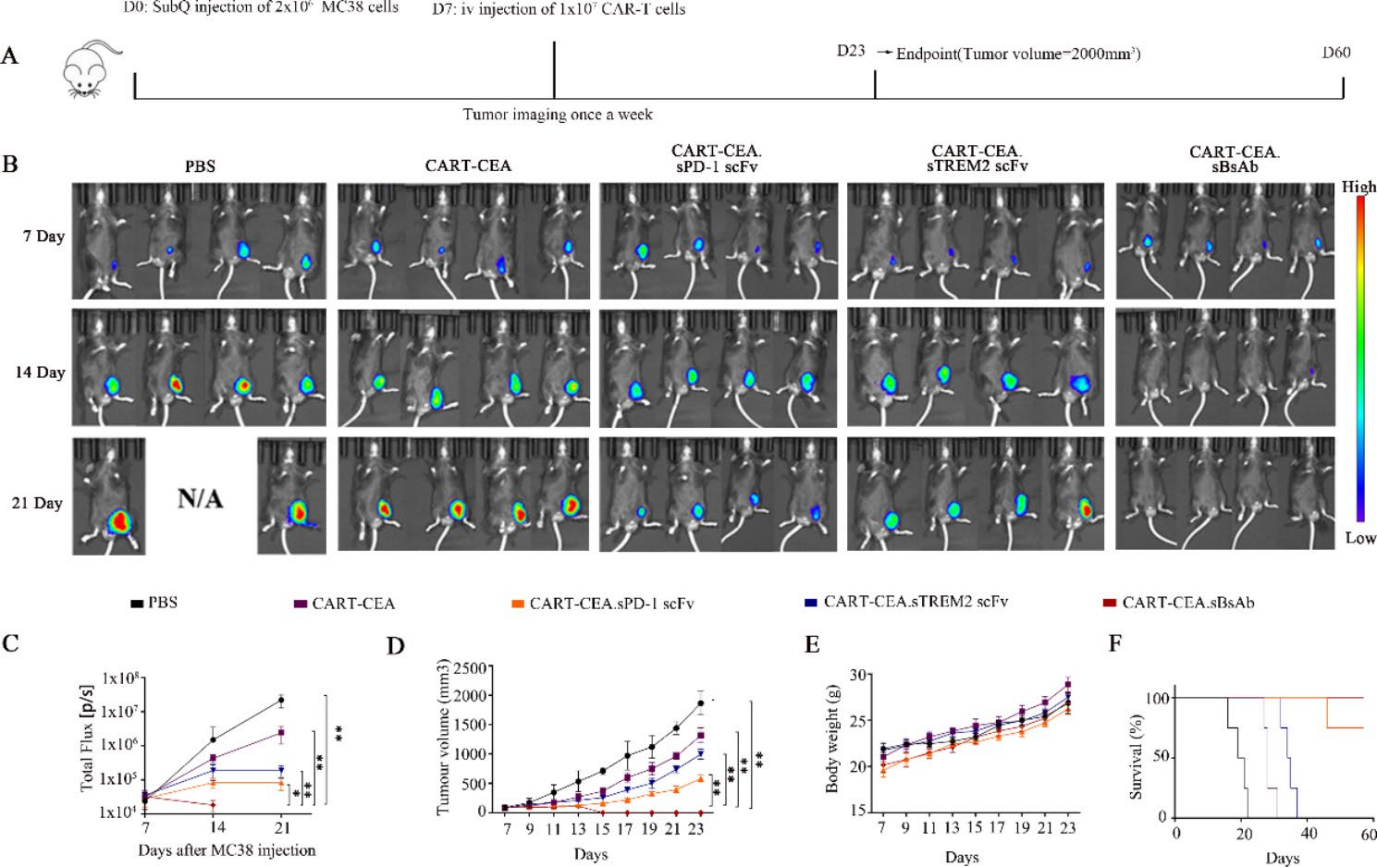
Anti-tumor effects of armed CAR-T cells in tumor-bearing mice. (**A**) Time-course diagram of the animal experiment. Six-week-old C57BL/6J mice were subcutaneously injected with 2 × 10^6^ MC38 cells expressing CEA. On day 7, mice from all groups were individually injected with 1 × 10^7^ constructed CAR-T cells through the tail vein. Tumor imaging was performed once a week. (**B**) Bioluminescence images of mice treated with CART-CEA, CART-CEA.sPD-1scFv, CART-CEA.sTREM2 scFv, and CART-CEA.sBsAb at days 7, 14, and 21; PBS treatment served as the control (n = 4). (**C**) Bioluminescence signals of mice in each group at days 7, 14, and 21 are shown on the Y-axis. The PBS group is shown in black, the CART-CEA group in purple, the CART-CEA.sPD-1 scFv group in orange, the CART-CEA.sTREM2 scFv group in blue, and the CART-CEA.sBsAb group in red. (**D**) Comparison of tumor volume over time in each group of tumor-bearing mice. (**E**) Weight development of mice during treatment in each group. (**F**) Effect of different treatments on the survival of tumor-bearing mice. Data are expressed as means ± SD; * *P* < 0.05; **P < 0.01, by unpaired Student’s t test. CAR-T, chimeric antigen receptor-modified-T; CEA, carcinoembryonic antigen; PD-1, programmed death-1; scFv, single-chain fragment variable; TREM2, triggering-receptor-expressed on myeloid cells 2; PBS, phosphate-buffered saline

Having successfully constructed BsAb-secreting CAR-T cells, the efficacy of CAR-T.sBsAb was further analyzed in the C57BL/6J mouse model with CRC. The experimental design is schematically presented in Fig. 5A. Significantly stronger and rapid inhibition of tumor progression was observed in the CART-CEA.sBsAb-treated group compared with that of the CART-CEA.sPD-1- and CART-CEA.sTREM2 scFv-treated CRC mice, as indicated by fluorescence intensity measurements (Fig. 5B-C). Consistent with these results, significantly greater inhibition of tumor growth was found in the CAR-T.sBsAb-treated mice than in the CART-CEA.sPD-1 scFv- and CART-CEA.sTREM2 scFv-treated groups, as indicated by the tumor growth curve (Fig. 5D **and Fig. S8A-E)**. There was no significant difference in the degrees of weight loss of mice among all treated groups (Fig. 5E). The survival of tumor-bearing mice was significantly enhanced in the CART-CEA.sBsAb-treated group compared with that in the other treatment groups (Fig. 5F). Taken together, these results confirmed the potent anti-tumor activity of CART-CEA.sBsAb compared with that of each element alone (CART-CEA.sPD-1 scFv and CART-CEA.sTREM2 scFv) in MC38 tumor-bearing mice. We also found that the tumor growth was more subdued in *Trem2–/–* mice than in wild-type mice (Fig. 5B-C **and Fig. S9A-C)**. The results were consistent with those of our previous study [20], Significantly stronger and rapid inhibition of tumor progression was observed in the CART-CEA.sBsAb-treated and CART-CEA.sPD-1-treated compared with that of the CART-CEA and CART-CEA.sTREM2 scFv-treated CRC mice, as indicated by fluorescence intensity measurements. Therefore, targeting TREM2 and PD-1 enhances the anti-tumor effects of CAR-T.

### BsAb secreting CAR-T cells remodeled the TME to achieve enhanced anti-tumor effects

**Fig. 6:**
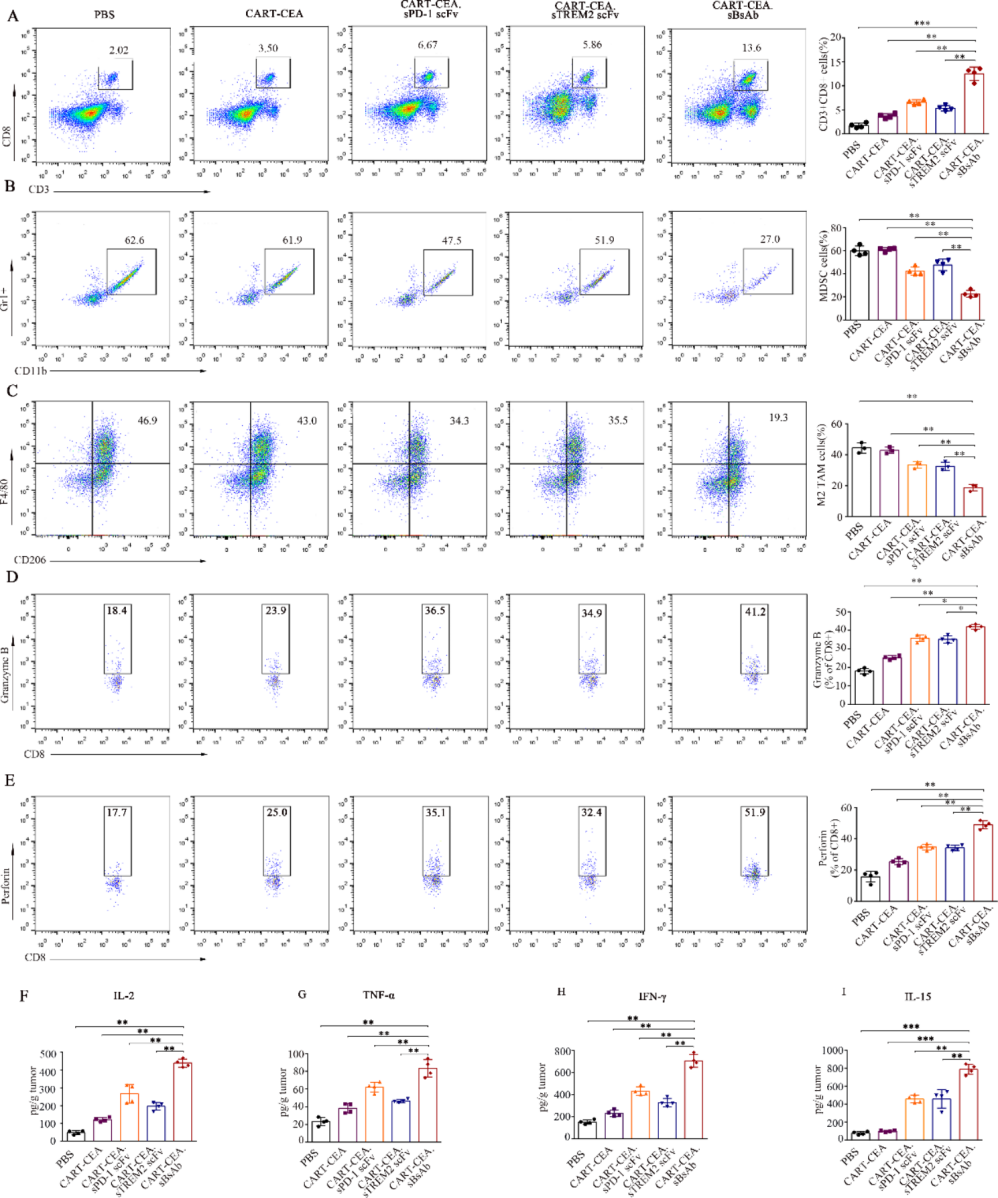
Adoptive transfer of bi-specific scFv antibody (BsAb)-secreting CAR-T cells modulate intratumoral cytokines and the proportions of infiltrating immune cells, MDSCs, and M2 TAMs. **(A)** Representative flow cytometry images and bar summary plots (right) showing the percentage of infiltrating CD8 + CD3 + T cells in tumor-bearing mice. Analysis of cell suspensions extracted from tumors by flow cytometry detecting infiltrating T cells originally gated as CD45 + cells. **(B-C)** Representative flow cytometry images and bar summary plots (right) showing the percentage of infiltrating MDSCs (**B**) and M2 TAMs **(C)** in tumor-bearing mice. Tumor cell suspensions were examined by flow cytometry to detect infiltration of MDSCs marked by CD11b + GR1 + and M2 TAMs marked by F4/80 + CD206+, initially gated as CD45 + cells. (**D-E**) Representative flow cytometry images and bar summary plots (right) showing the generation of perforin and granzyme B in tumor-infiltrating CD8-positive T cells. The percentages of CD8-positive T cells generating granzyme B (**D**) and perforin (**E**) in the TME were analyzed by flow cytometry. (**F-I**) Intratumoral cytokine evaluation of IL-12, TNF-α, IFN-γ, and IL-15 by ELISA. Data are expressed as means ± SD; * *P* < 0.05; **P < 0.01, by unpaired Student’s t test. scFv, single-chain fragment variable; CAR-T, chimeric antigen receptor-modified-T; MDSCs, myeloid-derived suppressor cells; TAMs, tumor associated macrophages; CD, cluster of differentiation; TME, tumor microenvironment; IL, interleukin; TNF, tumor necrosis factor; IFN, interferon; ELISA, enzyme-linked immunosorbent assay

The potential effect of CART-CEA.sBsAb on immune responses in the TME was analyzed based on the percentage of a parental population of CD8-positive T cells. As shown in Fig. 6A-C, significantly enhanced infiltration of CD8-positive T cells and CAR-T cells of CD8-positive T cells (Fig. 6A **and Fig. S10A-B)** along with decreased percentages of MDSCs (Fig. 6B) and M2 TAMs (Fig. 6C) were found in the tumor tissues of CART-CEA.sBsAb-treated mice compared with those of other groups. A recent study revealed that the inhibition of MDSCs and M2 TAMs suppressed not only CD8-positive T cells infiltration, but also the T cell response [31]. Accordingly, the effect of CART-CEA.sBsAb treatment in altering the immune response ability of CD8-positive T cells and CAR-T cells infiltration in tumor-bearing mice was further analyzed. The release of perforin and granzyme B by cytotoxic CD8-positive T cells and CAR-T cells was significantly enhanced in the CART-CEA.sBsAb-treated group compared with that in other treatment groups, as indicated by flow cytometry (Fig. 6D-E and **Fig. S11A-E**). Enhanced levels of the IL-2, IL-15, TNF-α, and IFN -γ were detected via ELISA in the intratumoral TME in the CAR-T.sBsAb-treated group compared with those in other groups (Fig. 6F-I), and accelerated proliferation of CAR-T cells was found following CART-CEA.sBsAb treatment **(Fig. S6H and Fig. S11A-B)**. Thus, targeting the TME decreased the accumulation of MDSCs and M2 TAMs, consequently alleviating the inhibition of CD8-positive T cells and CAR-T cells, and increasing their intratumoral proportion and cytotoxic ability in CART-CEA.sBsAb-treated mice. Importantly, infusion of CART-CEA.sBsAb T cells did not cause adverse effects the vital organs **(Fig. S13A)** or biochemical parameters of the mice **(Fig. S13B)**, confirming their safety for in vivo application.

## Discussion

In this study, we aimed to enhance the therapeutic effects of CAR-T therapy in solid tumors by reshaping the TME. Toward this end, we engineered CAR-T cells to secrete the bispecific PD-1-TREM2 scFv antibody into the TME, simultaneously targeting the checkpoint molecular PD-1 pathway, TAMs, and MDSCs with high expression of TREM2. Using a CRC mouse model, we demonstrated that the CAR-T cells engineered to secrete the BsAb PD-1-TREM2 scFv exhibited stronger anti-tumor potential than conventional CAR-T cells and CAR-T cells secreting PD-1 scFv alone. The mechanism of action for this enhanced therapeutic effect is based on the ability of PD-1-TREM2 scFv secreted by CAR-T cells into the TME to mitigate the immunosuppressive effects of the TME. Indeed, the PD-1-TREM2 scFv secreted by CAR-T cells reduced the suppressive effects of immune checkpoint molecule PD-1 and the accumulation of M2 TAMs and MDSCs in the TME. This phenomenon resulted in enhanced infiltration of immune T cells and CAR-T cells in the tumor tissue, increased in the release of perforin and granzyme B by T cells and CAR-T cells, and higher production of intra-tumor cytokines such as IFN-γ, TNFα, and IL-2, thereby potentiating tumoricidal activity.

Among adoptive cell transfer therapies, CAR-T cells are considered a novel therapeutic modality for alleviating cancer [32]. CAR-T therapy has achieved excellent success in alleviating hematologic malignancies, but its effect remains unsatisfactory in solid tumor treatment [33]. Unlike hematologic malignancies, solid tumors are characterized by the presence of a strong extracellular matrix (ECM) that obstructs the infiltration of CAR-T cells to the tumor interior[34]. Moreover, the tumoricidal efficacy of CAR-T cells in the tumor interior is inhibited by the TME, which consists of TAMs, myeloid suppressor immune cells, suppressive factors, an acidic environment, and overexpression of the immune checkpoint molecules PD-1/PD-L1 [35]. The application of CAR-T therapy to treat patients with CRC is currently in the clinical trial stage. The results of these trials have revealed that CAR-T treatment can alleviate the metastasis and development of CRC tumors; however, this effect is not consistent [9]. Immune checkpoint inhibitor (ICI) therapy targeting PD-1/PD-L1 has been shown to enhance the anti-tumor effects of CAR-T treatment [36]. However, the clinical benefits of combined ICI and CAR-T cell therapy are limited in treating patients with CRC, as less than 5% of patients respond to ICI therapy [37]. Several studies have reported that the proportion of intratumoral TAMs inversely correlates with the survival and recurrence in patients with solid tumors [38, 39]. TAMs directly or indirectly suppress the function of tumor-infiltrating CD8-positive T cells, secrete IL-10 cytokines to trigger immunosuppression, and support tumor cell proliferation and metastasis by promoting angiogenesis and ECM deposition[40]. Thus, targeting TAMs in the TME together with immune checkpoint therapy-enhanced CAR-T is a promising therapeutic strategy for alleviating CRC.

TREM2, a pattern recognition receptor, is a major immune signaling hub for the induction of disease pathophysiology[41]. TREM2 is involved in the pathogenesis of several diseases, including AD, multiple neurodegenerative diseases, metabolic syndrome-related obesity, fatty liver, and atherosclerosis [42]. In AD, TREM2 plays a protective role in the pathophysiological disease process by maintaining immune homeostasis in the brain, promoting the clearance of tissue debris, and abrogating potentially validated responses by binding to ligands such as ligand Aβ, apolipoprotein E, and plaque-associated lipids [16]. Accumulating evidence indicates a role of TREM2 in TAMs and MDSCs with immunosuppressive effects [18]. TREM2 expression is significantly upregulated in TAMs in the lungs of patients with cancer compared to that in healthy controls. Additionally, TREM2 levels in peritumor macrophages positively correlate with tumor progression [43], while TREM2-positive myeloid cells exhibit strong inhibitory effects against the proliferation of T cells in vitro [20]. Furthermore, TREM2 deficiency and anti-TREM2 monoclonal antibody treatment reportedly inhibit tumor growth in mice with CRC and ovarian cancer. Notably, the efficiency of anti-PD-1 treatment was enhanced when TREM2 was absent or activated by monoclonal antibodies [20]. In the present study, we further demonstrated that treatment with the BsAb PD-1-TREM2 scFv, yielded significant anti-tumor effects compared to those of a single treatment targeting PD-1, which was achieved by reducing the tumor infiltration of MDSCs and TAMs and increasing the production of tumor-infiltrating T cells. These results are consistent with those of previous studies. PD-1-TREM2 scFv prevented TAMs and myeloid suppressor cells from suppressing the functions of T-cells and increased the release of perforins and granzyme B from cytotoxic T cells. However, specific targeting of TREM2 had no effect other than decreasing the ratio of TAMs and MDSCs. The mechanism underlying this effect remains to be elucidated. TREM2 has primary been shown to be expressed on the surface of cells in the tumor associated macrophage (TAM) and tumor-infiltrating myeloid cells, no TREM2 was detected in DCs or lymphoid cells in tumor microenvironment (TME) [20, 44]. Tumor-infiltrating myeloid cells include both immunostimulatory and immunosuppressive subsets. Immunostimulatory myeloid cells include type 1 dendritic cells (DC1s) and M1-like macrophages; suppressive myeloid cells include M2-like macrophages, as well as a heterogeneous group of myeloid progenitor cells and immature myeloid cells collectively defined as myeloid-derived suppressor cells (MDSCs)[44]. By targeting TREM2 on MDSCs, we anticipate the potential blockade of their pro-tumoral functions or restoration of DCs’ immunostimulatory properties, thereby augmenting the immune cells’ response.

BsAb therapy has led to significant advances in tumor treatment [45]. Targeting of CD19 and CD3 in acute lymphoblastic leukemia formed the basis for the world’s first FDA-approved BsAb-based immunotherapy [46]. BsAb are structurally similar to monoclonal antibodies, containing two scFvs derived from monoclonal antibodies linked with a flexible peptide [47]. Therefore, BsAb have a lower molecular weight and greater tissue permeability than monoclonal antibodies [21]. Due to the lack of Fc segments, BsAb do not induce the same adverse effects as monoclonal antibodies in tumor treatment, which includes nonspecific involvement of diverse organs and the development of immune-related adverse reactions in patients [48, 49]. However, the low molecular weight of BsAb results in their rapid clearance from the body, warranting the need for vectors capable of extending their half-life [50, 51]. Previously, researchers have designed human macrophages engineered to secrete a bi-specific scFv antibody in support of antigen-dependent T cell responses to glioblastoma [52]. However, the applicability of this treatment method is limited, as macrophages engineered to secrete a BsAb without tumor targeting cannot accumulate at tumor sites. To address this issue, we engineered CAR-T cells to secrete the BsAb PD-1-TREM2 scFv, as these cells can target tumor sites and continuously secrete and lead to the accumulation of BsAb at tumor sites. Thus, this therapeutic approach not only overcomes the limitations imposed by the short half-life of BsAb enabling a reduction in BsAb treatment frequency, but also the PD-1-TREM2 scFv secreted by CAR-T cells can further alleviate the immunosuppressive effects of the TME, thereby enhancing the anti-tumor efficacy of existing CAR-T cell treatment.

## Conclusions

In this study, we combined CAR-T cells and BsAb into a single immunotherapeutic platform. Our results showed that CAR-T cells secreted PD-1-TREM2 scFV into the TME and that this approach resulted in superior anti-tumor efficacy relative to the treatment strategy involving CAR-T cells that secrete PD-1 scFv alone. Therefore, the engineering of CAR-T cells to secrete a BsAb targeting the TME may improve the clinical efficacy of CAR-T cells and checkpoint inhibitor immunotherapy.

### Electronic Supplementary Material

Below is the link to the electronic supplementary material.

Supplementary Material 1

## Data Availability

The datasets used and/or analyzed during the current study are available from the corresponding author on reasonable request.

## Abbreviations

CAR: Chimeric antigen receptor
PD-1: Programmed death-1
TREM2: Triggering-receptor-expressed on myeloid cells 2
TME: Tumor microenvironment
TAMs: tumor-associated macrophages
MDSCs: Myeloid-derived suppressor cells
CEA: Carcinoembryonic antigen
scFv: Single-chain variable fragment
CRC: Colorectal cancer
AD: Alzheimer’s disease
BMDMs: Bone-marrow-derived macrophages
CHO: Chinese hamster ovary
SDS-PAGE: Sodium dodecyl-sulfate polyacrylamide gel electrophoresis
APOE: Apolipoprotein E
BsAb: Bi-specific scFv antibody
ECM: Extracellular matrix
IL-2: Interleukin-2
TNF-α: Tumor necrosis factorα
IFN-γ: interferon-γ
ELISA: Enzyme-linked immunosorbent assay
