# Supplementary material for: Target delivery of a PD-1-TREM2 scFv by CAR-T cells enhances anti-tumor efficacy in colorectal cancer
Source: Mol Cancer. 2023 Aug 10;22:131. doi: 10.1186/s12943-023-01830-x (PMC10413520; doi:10.1186/s12943-023-01830-x)

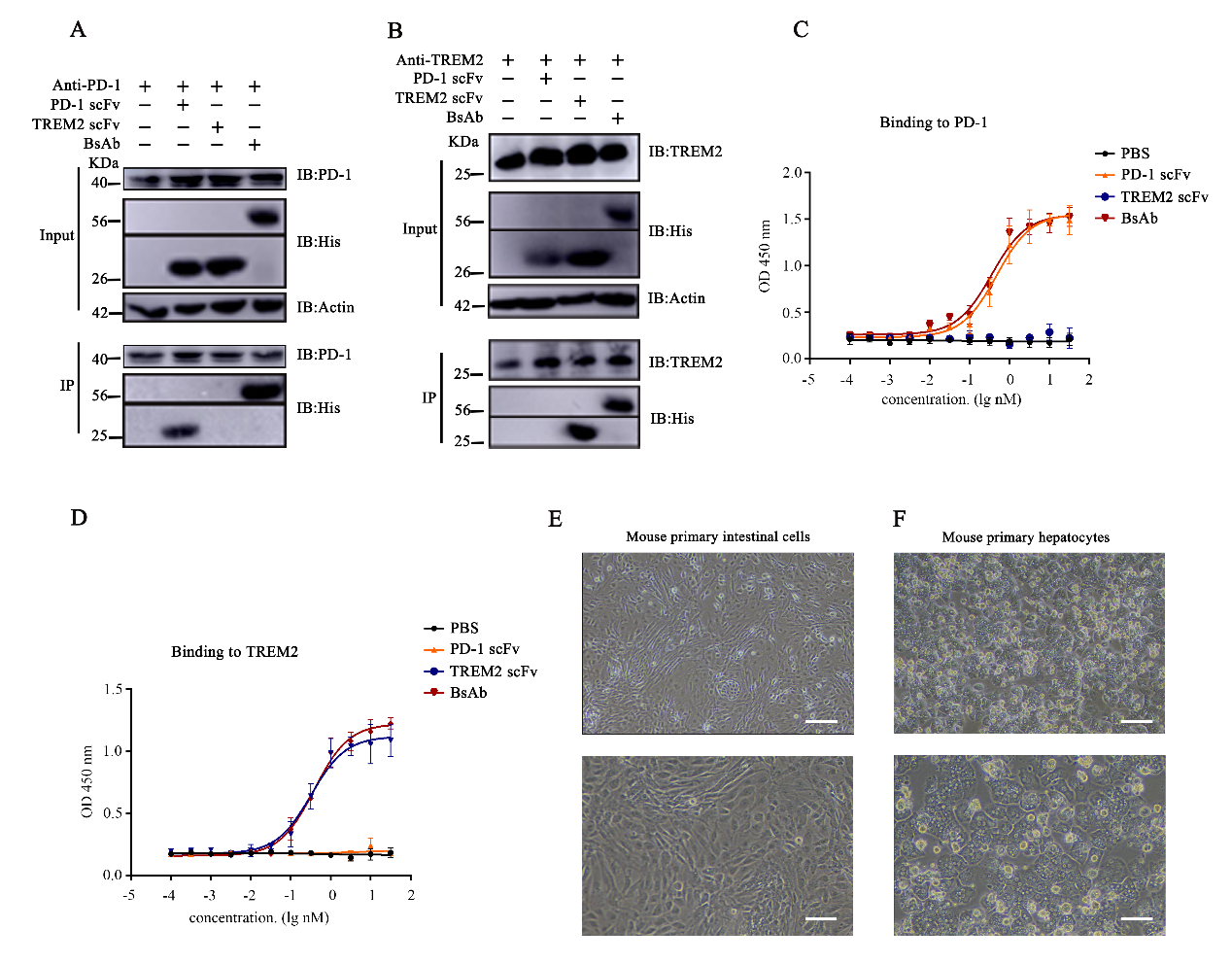


**Figure S1.** Functional characteristics of PD-1scFv, TREM2 scFv and BsAb. **(A-B)** Interaction between PD-1scFv, TREM2 scFv, and BsAb on HEK-293T-PD-1- myc-tagged cells and TREM2 on RAW 264.7 cells examined by co-immunoprecipitation (IP) and immunoblotting (IB). **(C-D)** Binding ability of PD-1scFv, TREM2 scFv, and BsAb to PD-1 and TREM2 using sandwich ELISA, wherein PD-1scFv, TREM2 scFv, and BsAb were added to PD-1 or TRME-2 coated plates, followed by detection by anti-His-tag antibody conjugated with HRP. **(E-F)** Representative images showing the status of mouse primary intestinal cells **(E)** and mouse primary hepatocytes **(F)** incubated with BsAb. Scale bar: 200μm (upper) and 100μm (lower). PD-1, programmed death-1; scFv, single-chain fragment variable; TREM2, triggering-receptor-expressed on myeloid cells 2; HRP, horseradish peroxidase; RAW 264.7, mouse leukemia cells of monocyte macrophage.


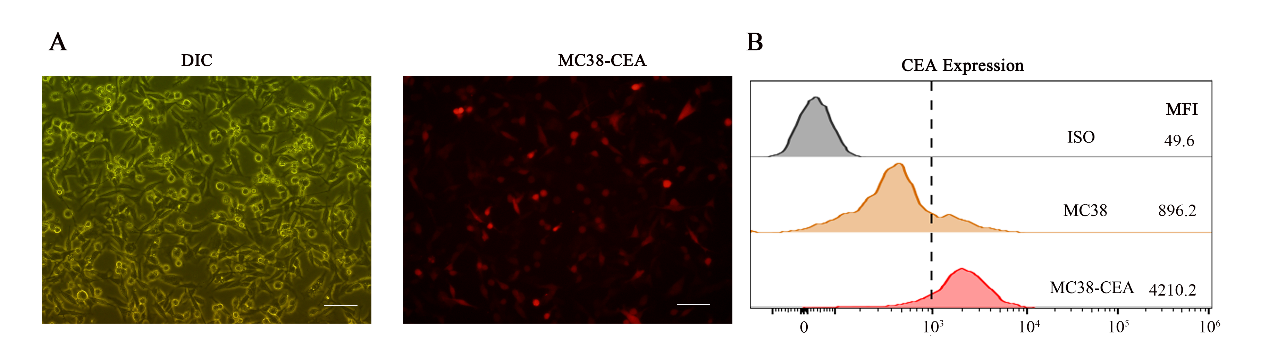


**Figure S2.** Preparation of the MC38 cell line stably expressing CEA**.** (**A**) Representative fluorescent images showing expression of CEA (red) in MC38 cells by lentiviral infection. Scale bar: 50μm. (**B**) Flow cytometric histograms representing CEA expression in MC38 cells for lentiviral infection. Control (black), MC38 (orange), and MC38-CEA (red).


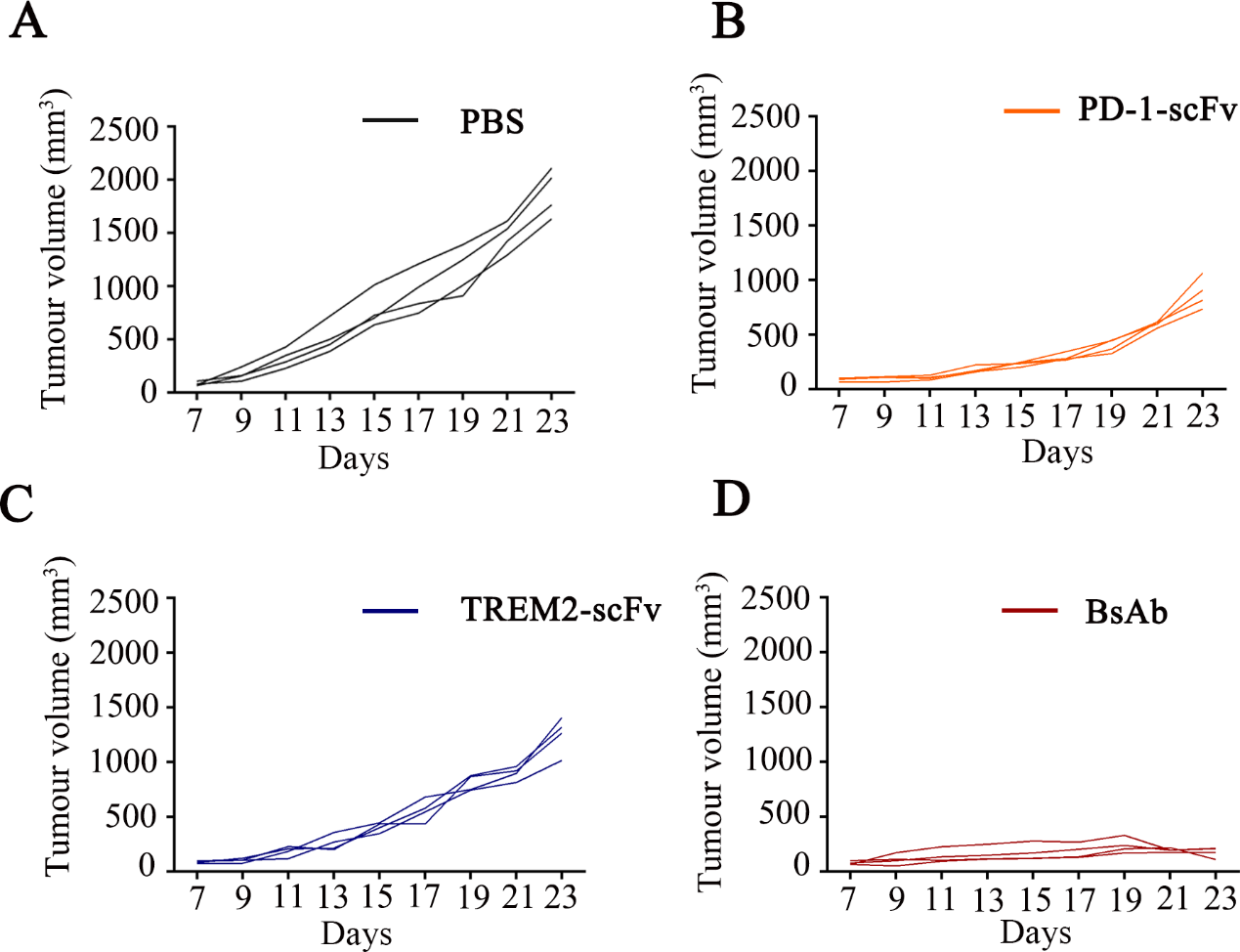


**Figure S3.** Anti-tumor function of PD-1 scFv, TREM2 scFv, and BsAb**.** (**A-D**) Growth curves of individual tumors in MC38-CEA tumor-bearing mice infused with PBS (black line) (**A**), PD-1 scFv (orange line) (**B**), TREM2 scFv (blue line) (**C**), or BsAb (red line) (**D**); n = 4 per group.


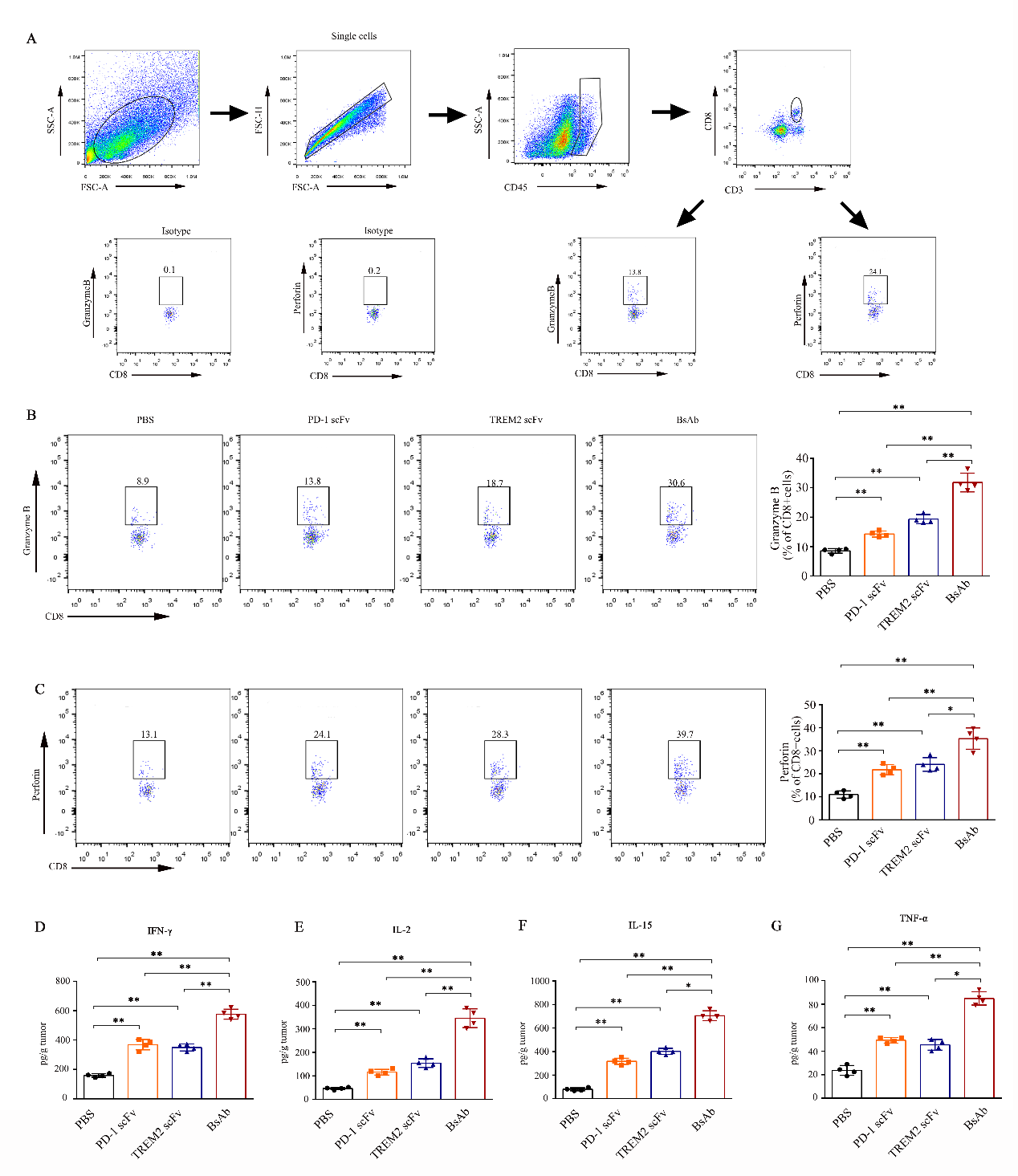


**Figure S4. The generation of intratumoral cytokines and the release granzyme B/perforin of CD8 + T cells in treated mice. (A)** Representative flow cytometry images of the gating strategy for granzyme B, perforin, and isotype. **(B-C)** Representative flow cytometry images and bar summary plots (right) showing the generation of perforin and granzyme B in tumor-infiltrating CD8-positive T cells. The percentages of CD8-positive T cells generating granzyme B (**B**) and perforin (**C**) in the TME were analyzed by flow cytometry. (**D-G**) Intratumoral cytokine evaluation of IL-12, TNF-α, IFN-γ, and IL-15 by ELISA. Data are expressed as means ± SD, * *P* < 0.05; ***P* < 0.01. scFv, by one-way ANOVA. single-chain fragment variable; CD, cluster of differentiation; TME, tumor microenvironment; IL, interleukin; TNF, tumor necrosis factor; IFN, interferon; ELISA, enzyme-linked immunosorbent assays.


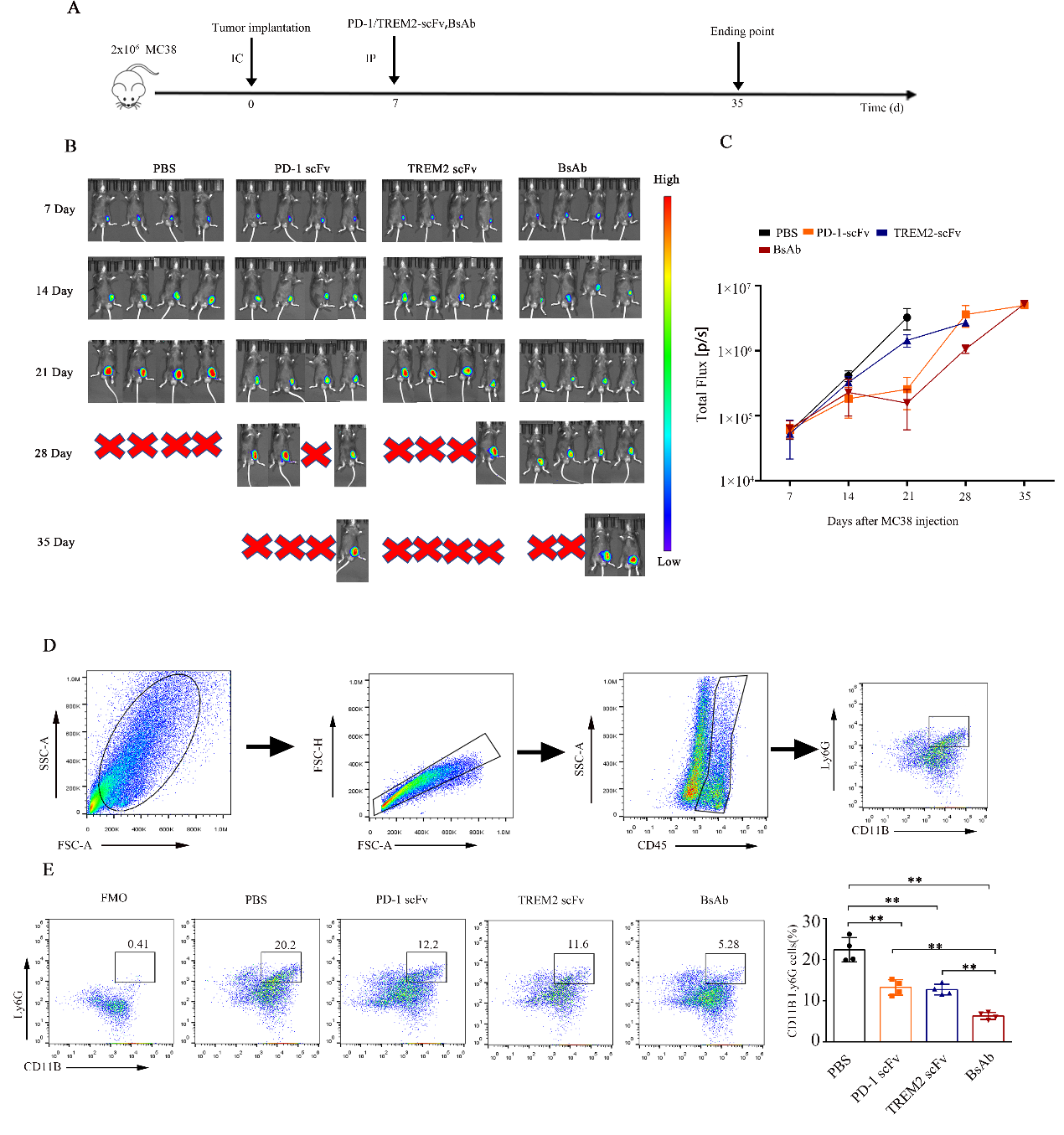


**Figure S5.** **Tumor recurrence in BsAb-treated mice**

**(A)** Schematic representation of the animal experiment. Six-week-old C57BL/6J mice were subcutaneously injected with 2 ×10^6^ MC38 cells expressing CEA. On day 7, mice received intravenous injection of PD-1 scFv, TREM2 scFv and BsAb at 200 µg/mouse daily; the PBS-treated group served as the control. (B) Bioluminescence images of mice treated with PD-1 scFv, TREM2 scFv, and BsAb at day 7, 14, 21, 28 and 35 (n = 4). (C) Bioluminescence signals of individual groups at day 7, 14, 21, 28 and 35 are shown on the Y-axis. The PBS group is indicated as black, PD-1 scFv group is in orange, TREM2 scFv group is in blue, and the BsAb group is in red. **(D)** Representative flow cytometry images of gating strategy of MDSC (CD11B + Ly6G). **(E)** Representative flow cytometry images and bar summary plots (right) showing the generation of MDSCs (CD11B + ly6G+) in the TME analyzed by flow cytometry. Data are expressed as means ± SD, * *P* < 0.05; ***P* < 0.01. scFv, by one-way ANOVA. scFv, single-chain fragment variable; CEA, carcinoembryonic antigen; PD-1, programmed death-1; TREM2, triggering-receptor-expressed on myeloid cells 2; PBS, phosphate buffered saline; MDSCs, myeloid-derived suppressor cells; TAMs, tumor associated macrophages; CD, cluster of differentiation.


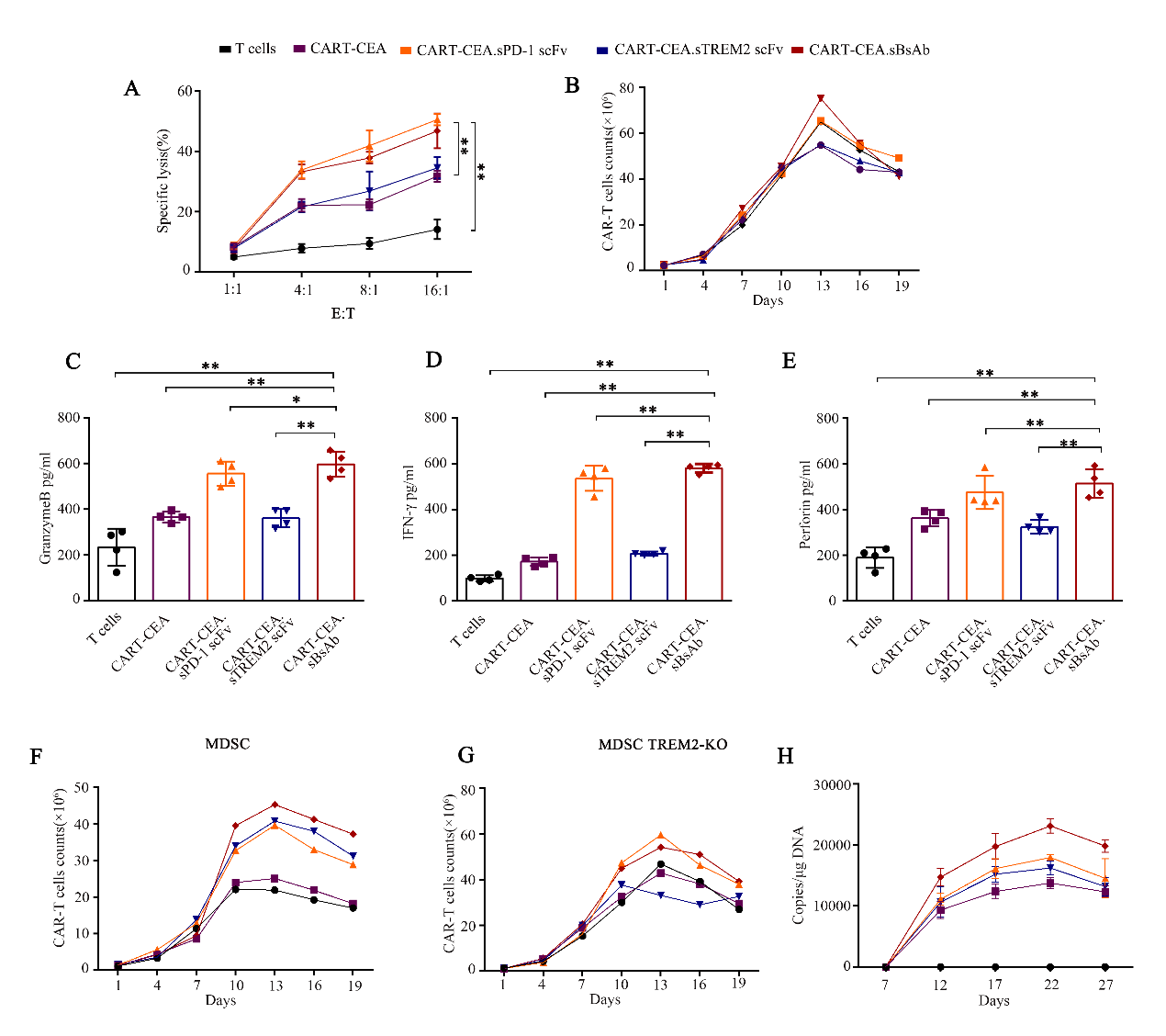


**Figure S6. Survival, proliferation, and functional of engineered CAR-T cells**

**(A)**Cytotoxicity of all constructed CAR-T cells against MC38 cells. All groups of CAR-T cells were co-cultured with MC38 cells at 1:1, 4:1, 8:1, and 16:1, ratios, and MC38 cells lysis was detected by Cr51 release assay. **(B)** Survival and proliferation of engineered CAR-T cells by count at day1, 4, 7, 10, 13, 16, and 19. **(C-E)** Detection of cytokines produced by CAR-T cells with ELISA. **(F)** Survival and proliferation of engineered CAR-T cells in co-culture with wild-type MDSCs (CD11B + GR1 + sorting) at a 1:1ratio by count at days 1, 4, 7, 10, 13, 16, and 19. **(G)** Survival and proliferation of engineered CAR-T cells in co-culture with TREM2-knockout (KO) MDSCs by count at day1, 4, 7, 10, 13, 16, and 19. **(H)** Survival and proliferation of engineered CAR-T cells in vivo by PCR at days 7, 12, 17, 22, and 27. Data are expressed as means ± SD, * *P* < 0.05; ***P* < 0.01, by unpaired Student’s t test. scFv, single-chain fragment variable; MDSCs, myeloid-derived suppressor cells; IFN, interferon; ELISA, enzyme-linked immunosorbent assay.


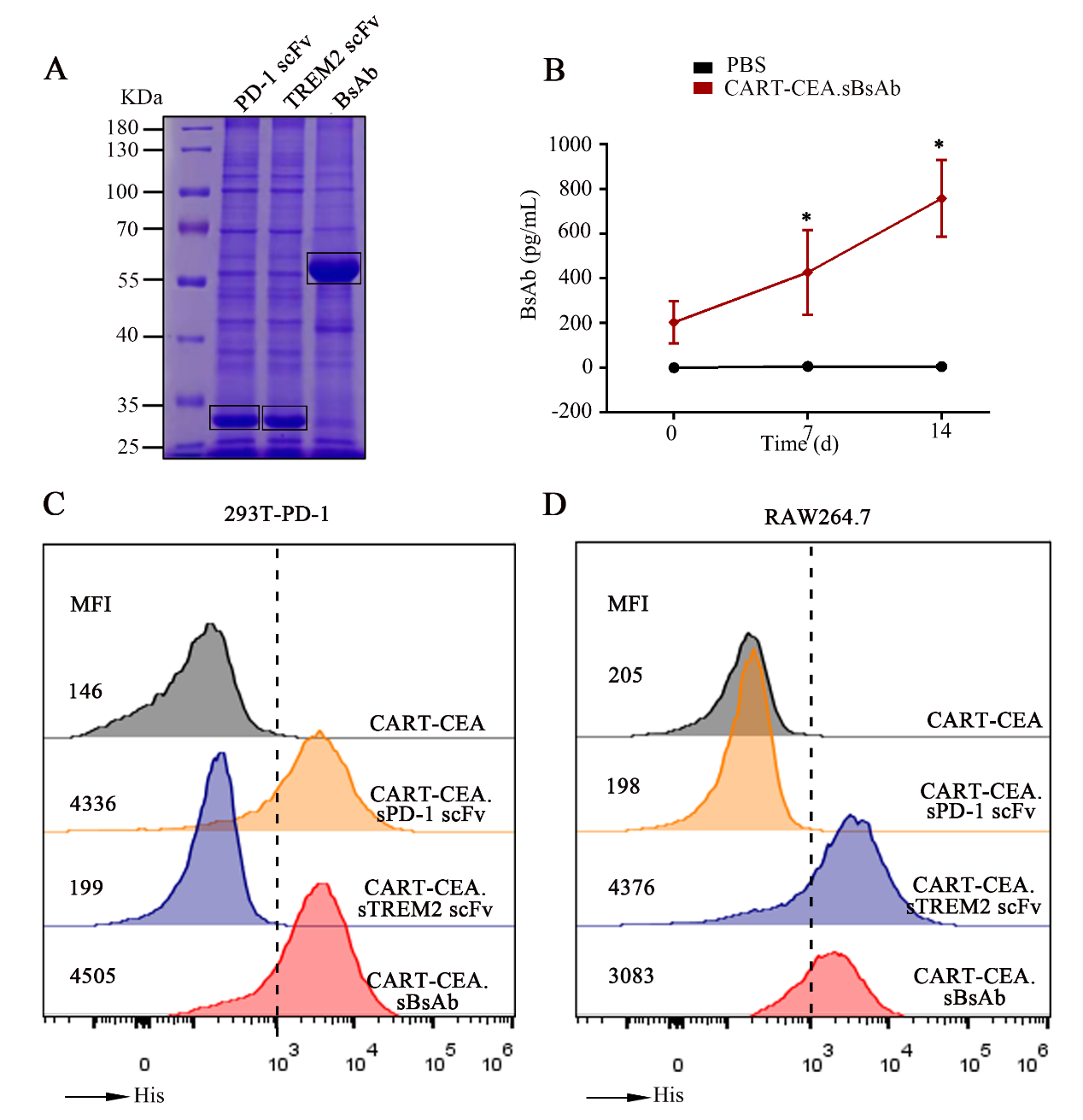


**Figure S7. PD-1 scFv, TREM2 scFv, and BsAb are secreted by CAR-T cells and conjugated to specifically targeted antigens.**

**(A**) SDS-PAGE analysis of supernatants produced by genetically modified CAR-T cells secreting PD-1 scFv, TREM2 scFv, and BsAb subjected to purification and concentration using a nickel column. Detection of PD-1 scFv, TREM2 scFv, and BsAb expression in the concentrated supernatant by SDS-PAGE. (**B**) The concentration of BsAb in the supernatant increased over time. Mouse T cells transduced with CART-CEA.sBsAb supernatants were harvested at days 0, 7, and 14 for His-tag ELISA. (**C-D**) Flow cytometric histograms demonstrating His-tag detection of CART-CEA.sPD-1 scFv, CART-CEA.sTREM2 scFv, and CART-CEA.sBsAb binding to 293T stably expressing PD-1 (293T-PD-1) **(C)** and TREM2 on the RAW 264.7 cells **(D)**. The 293T-PD-1 cells and RAW 264.7 cells were treated with the concentrated PD-1 scFv, TREM2 scFv, and BsAb secreted from CART-CEA.sPD-1 scFv, CART-CEA.sTREM2 scFv, and CART-CEA.sBsAb and MFI of His was detected by flow cytometry. Data are expressed as means ± SD, * *P* < 0.05; ***P* < 0.01, by unpaired Student’s t test. PD-1, programmed death-1; scFv, single-chain fragment variable; TREM2, triggering-receptor-expressed on myeloid cells 2; SD, standard deviation.


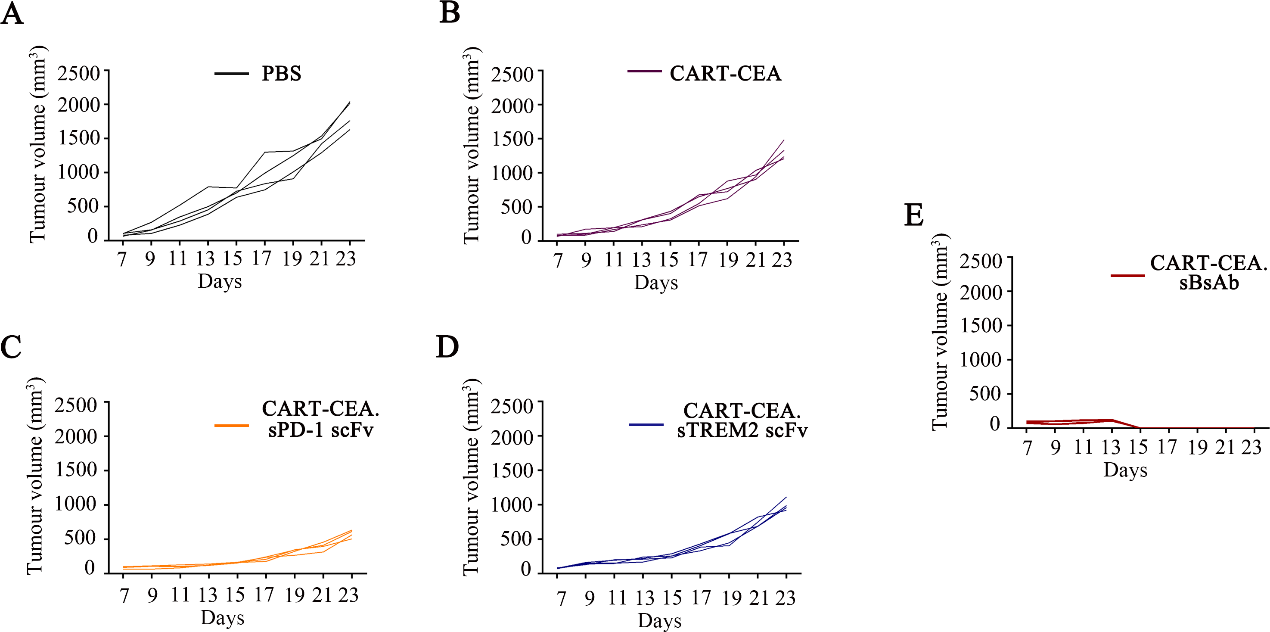


**Figure S8.** **Anti-tumor function of the constructed CAR-T cells.**

(**A-E**) Growth curve of individual tumors in MC38-CEA tumor-bearing mice infused with PBS (black line) (**A**), CART-CEA (purple line) (**B**), CART-CEA.sPD-1 scFv (orange line) (**C**), CART-CEA.sTREM2 scFv(blue line) (**D**) or CAR-CEA.sBsAb(red line) (**E**); n = 4 per group. PD-1, programmed death-1; scFv, single-chain fragment variable; TREM2, triggering-receptor-expressed on myeloid cells 2.


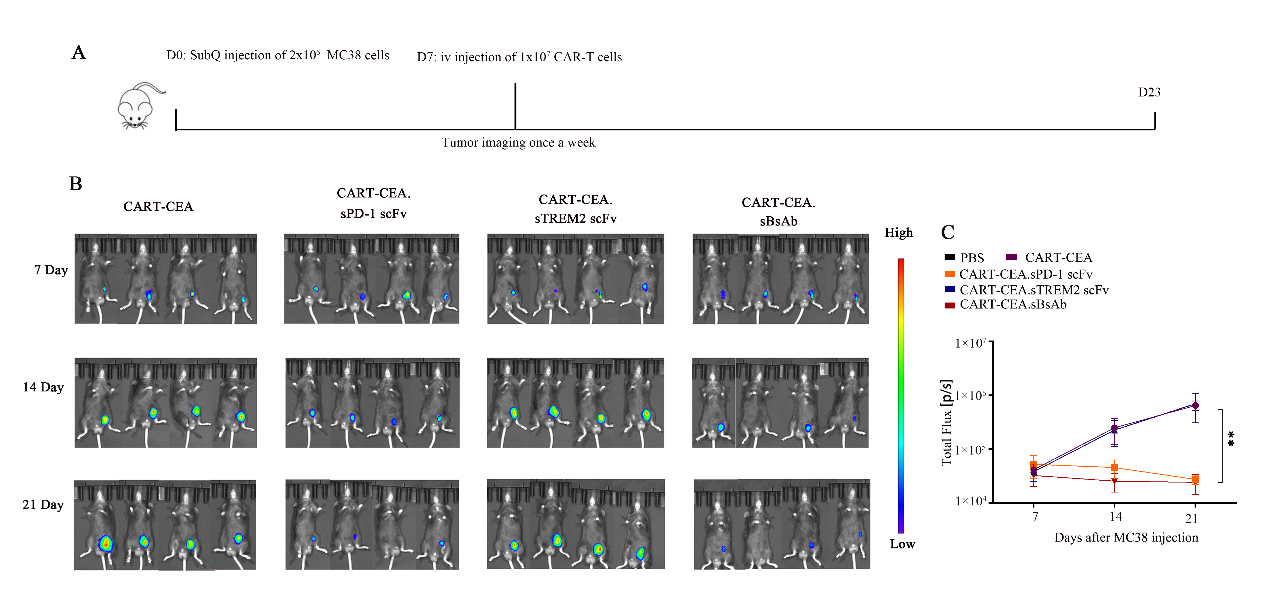


**Figure S9.** **Anti-tumor function of the constructed CAR-T cells in *TREM2^–/–^* mice.**

(A) Time-course diagram of the animal experiment. Six-week-old *TREM2^–/–^* C57BL/6J mice were subcutaneously injected with 2 × 10^6^ MC38 cells expressing CEA. On day 7, mice from all groups were individually injected with 1 × 10^7^ constructed CAR-T cells through the tail vein. Tumor imaging was performed once a week. (B) Bioluminescence images of mice treated with CART-CEA, CART-CEA.sPD-1scFv, CART-CEA.sTREM2 scFv, and CART-CEA.sBsAb at days 7, 14, and 21; PBS treatment served as the control (n = 4). (C) Bioluminescence signals of mice in each group at day 7, 14, and 21 are shown on the Y-axis. The PBS group is shown in black, CART-CEA group in purple, CART-CEA.sPD-1 scFv group in orange, CART-CEA.sTREM2 scFv group in blue, and CART-CEA.sBsAb group in red. Data are expressed as means ± SD, * *P* < 0.05; ***P* < 0.01, by unpaired Student’s t test. CAR-T, chimeric antigen receptor-modified-T; CEA, carcinoembryonic antigen; PD-1, programmed death-1; scFv, single-chain fragment variable; TREM2, triggering-receptor-expressed on myeloid cells 2; PBS, phosphate-buffered saline.


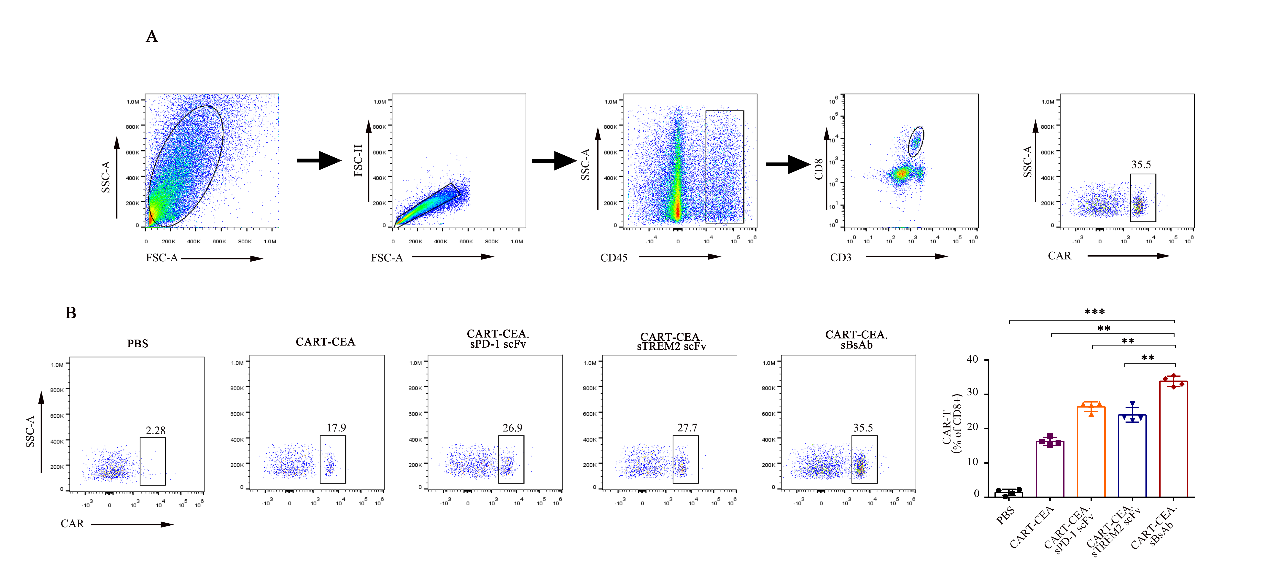


**Figure S10. Adoptive transfer of bi-specific scFv antibody (BsAb)-secreting CAR-T cells modulate the percentage of infiltrating CAR-T cells.**

**(A)**Representative flow cytometry images of the gating strategy for identifying CAR-T cells among CD8 + T cells. **(B)** Representative flow cytometry images and bar summary plots (right) showing the generation of CAR-T cells from CD8 + T cells in the TME analyzed by flow cytometry. Data are expressed as means ± SD, * *P* < 0.05; ***P* < 0.01, by unpaired Student’s t test. CAR-T, chimeric antigen receptor-modified-T; CEA, carcinoembryonic antigen; PD-1, programmed death-1; scFv, single-chain fragment variable; TREM2, triggering-receptor-expressed on myeloid cells 2; PBS, phosphate-buffered saline.


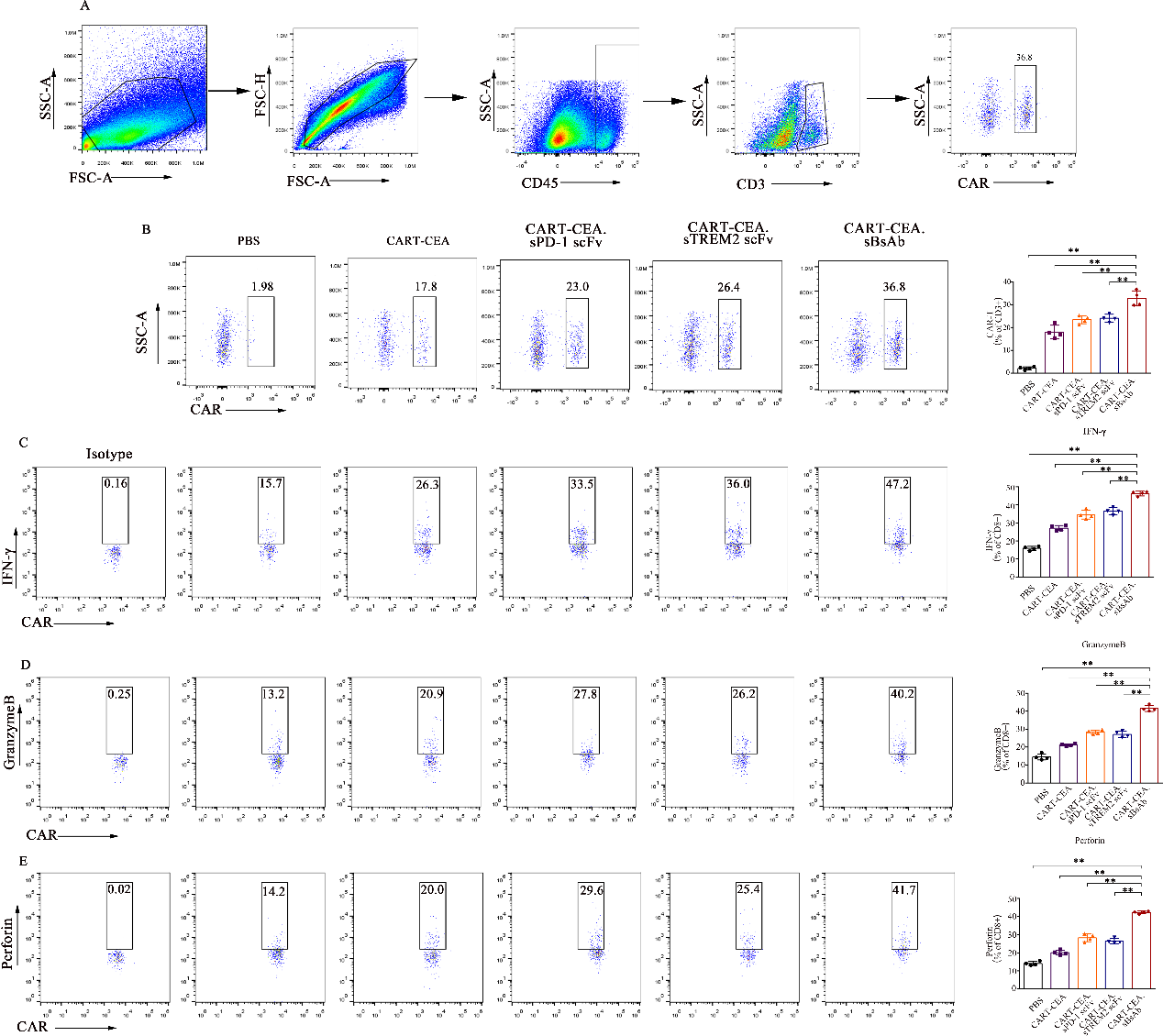


**Figure S11. Release of IFN-γ, granzyme B, and perforin by CAR-T cells in the intratumor environment.**

**(A**) Representative flow cytometry images of the gating strategy for identifying CAR-T cells, IFN-γ, granzyme B, and perforin. **(B)** Representative flow cytometry images and bar summary plots (right) showing the generation of CAR-T cells in the TME analyzed by flow cytometry. **(C-E)** Representative flow cytometry images and bar summary plots (right) showing the release of IFN-γ, granzyme B, and perforin of CAR-T cells in the TME analyzed by flow cytometry. Data are expressed as means ± SD, * *P* < 0.05; ***P* < 0.01, by unpaired Student’s t test. scFv, single-chain fragment variable; CAR-T, chimeric antigen receptor-modified-T; CD, cluster of differentiation; IFN, interferon.


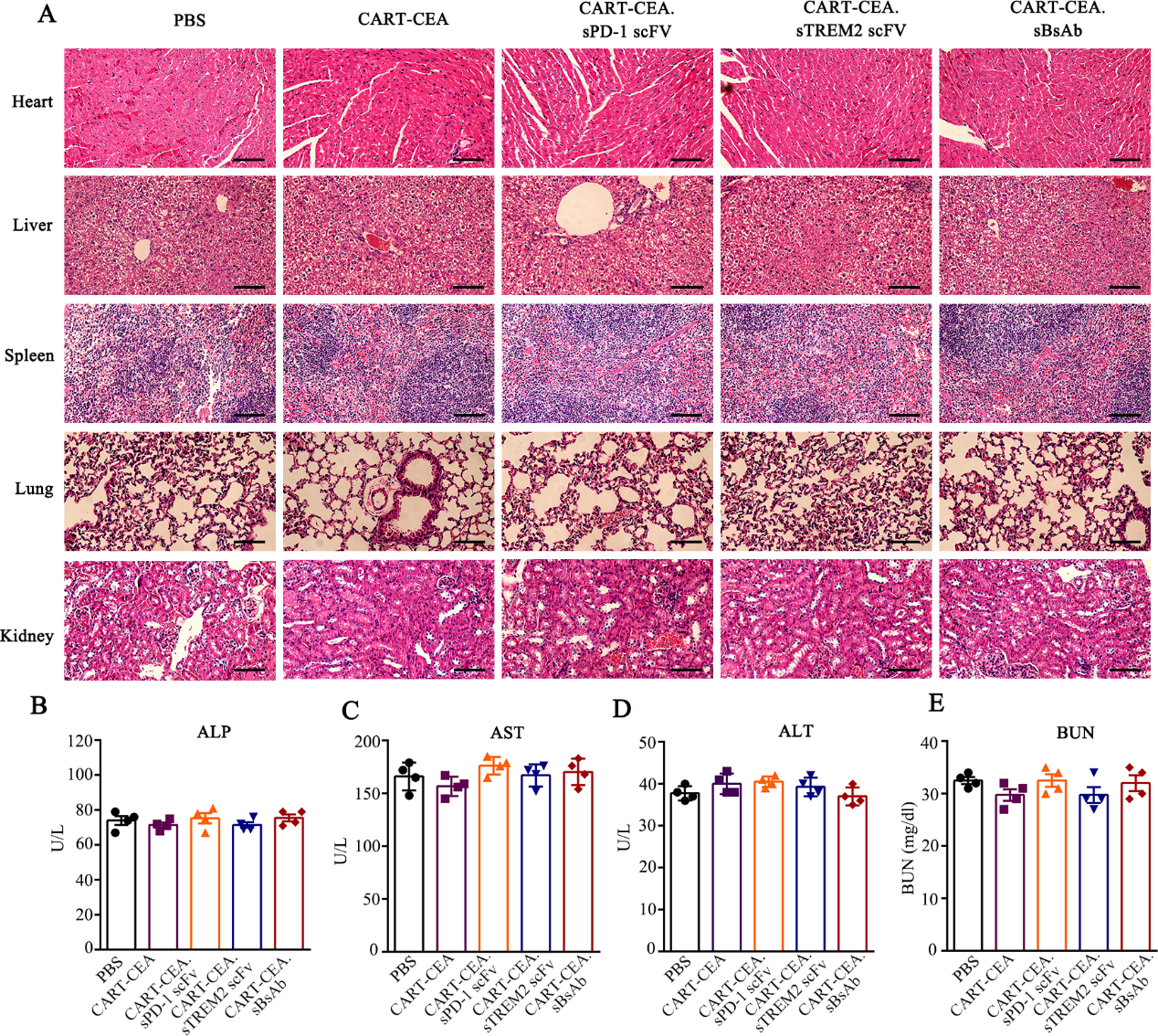


**Figure S12. Biosafety evaluation of the constructed CAR-T cells.**

1. Histological evaluation of major organs, namely the heart, liver, spleen, lung, and kidney of mice by HE staining. Scale bars: 50μm. (**B-F**) Hematologic markers representing the liver (ALT, AST, ALP) and kidney (BUN) function in healthy C57BL/6J mice infused with PBS, CART-CEA, CART-CEA.sPD-1 scFv, CART-CEA.sTREM2 scFv, or CART-CEA.sBsAb. Serum was collected at the termination of animal experiments. CAR-T, chimeric antigen receptor-modified-T; HE, hematoxylin and eosin; ALT, alanine transaminase; AST, aspartate transaminase; BUN, blood urea nitrogen; PBS, phosphate buffered saline; CEA, carcinoembryonic antigen; PD-1, programmed death-1; TREM2, triggering-receptor-expressed on myeloid cells 2.


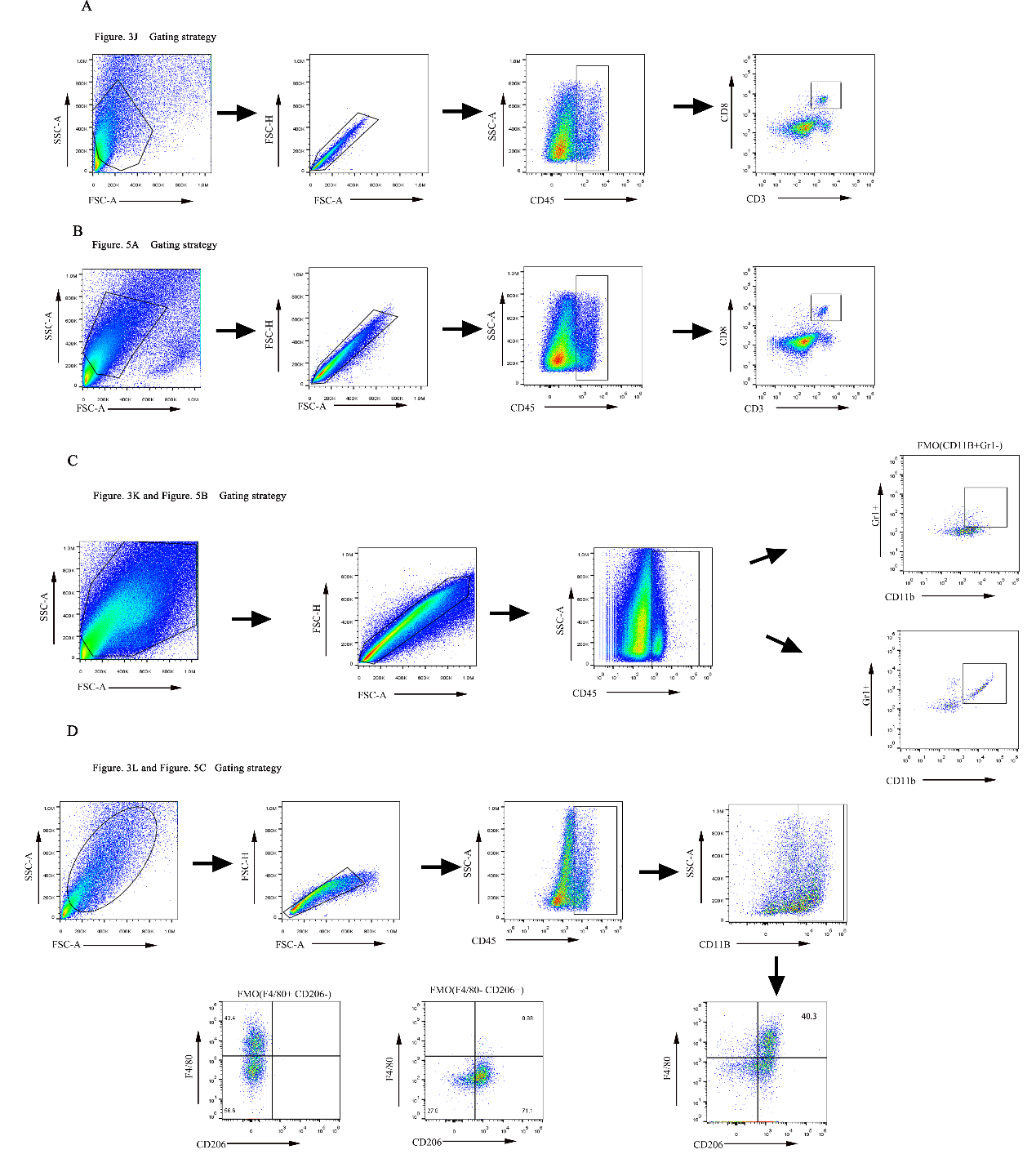


**Figure S13. Gating strategy.**

**(A)** Representative flow cytometry images of the gating strategy for identifying CD8^+^ T cells in Fig. 3J. **(B)** Representative flow cytometry images of the gating strategy for identifying CD8^+^ T cells in Fig. 5A. **(C)** Representative flow cytometry images of the gating strategy for identifying MDSCs in Fig. 3K and Fig. 5B. **(D)** Representative flow cytometry images of the gating strategy and FMO of TAM cells in Fig. 3L and Fig. 5C. CD, cluster of differentiation;


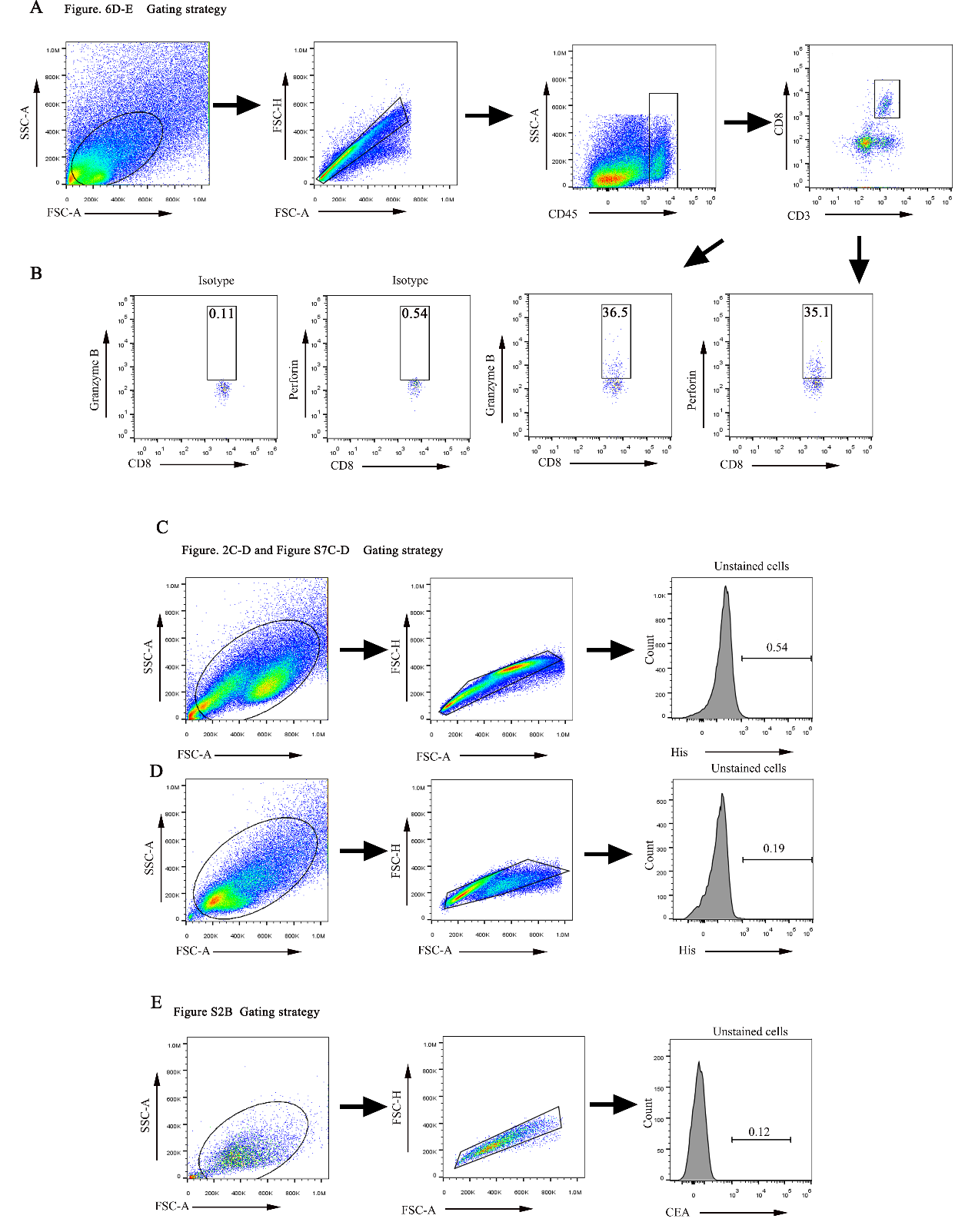


**Figure S14. Gating strategy.**

**(A-B)** Representative flow cytometry images of the gating strategy and isotype of granzyme B and perforin in Fig. 6D-E. **(C-D)** Representative flow cytometry images of gating strategy of the His expression on HEK-293T-PD1 cells and RAW 264.7 cells in Fig. 2C-D and Figure S7C-D. **(C)** Representative flow cytometry images of the gating strategy of MC38-CEA in Figure S2B. CD, cluster of differentiation.


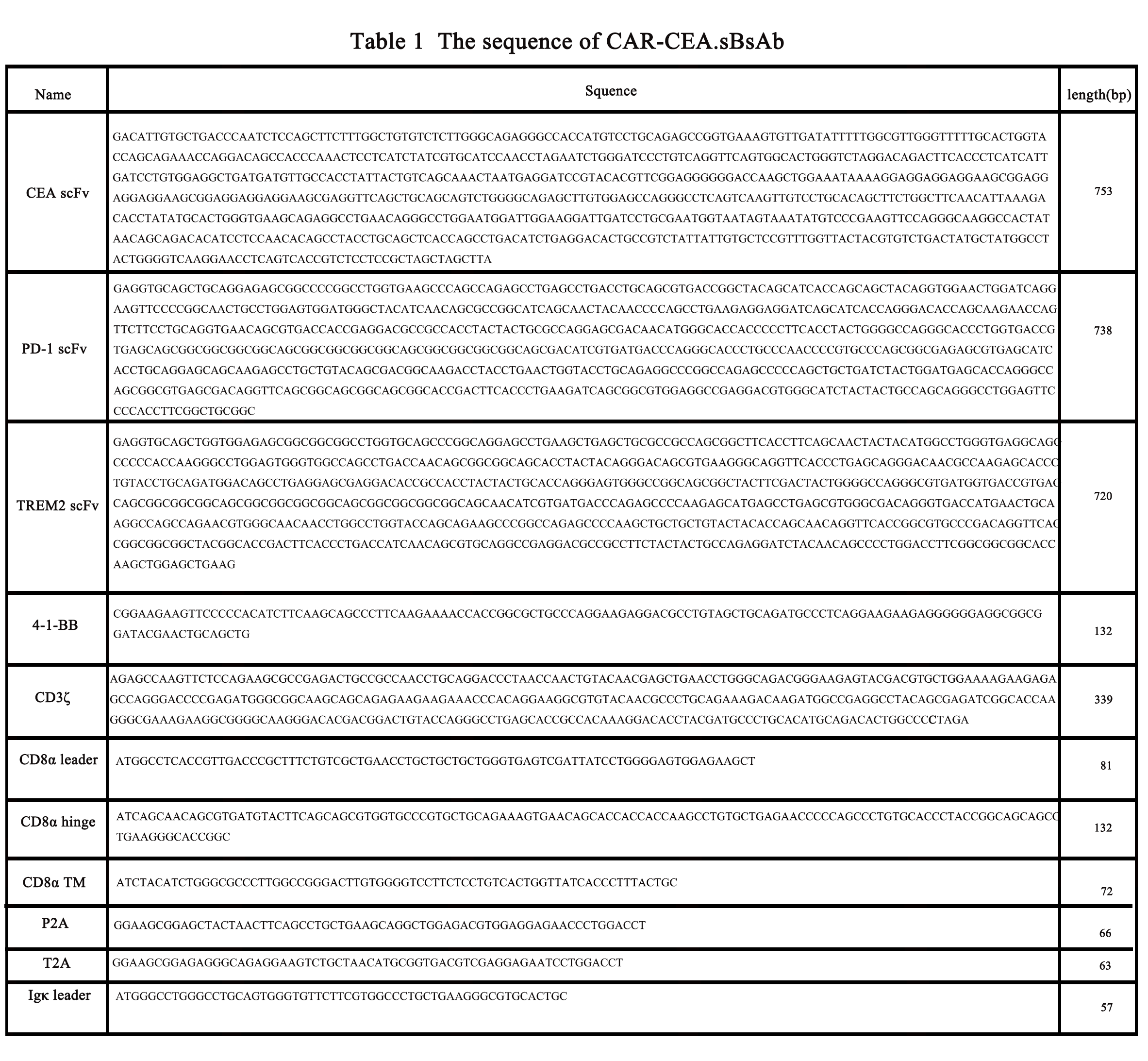

Supplement: Supplementary file 1 — Supplementary Material 1 [file 12943_2023_1830_MOESM1_ESM.docx]
